# Senescence-associated IL-6 and IL-8 cytokines induce a self- and cross-reinforced senescence/inflammatory milieu strengthening tumorigenic capabilities in the MCF-7 breast cancer cell line

**DOI:** 10.1186/s12964-017-0172-3

**Published:** 2017-05-04

**Authors:** Paola Ortiz-Montero, Arturo Londoño-Vallejo, Jean-Paul Vernot

**Affiliations:** 1 Cellular and Molecular Physiology Group, Instituto de Investigaciones Biomédicas, Facultad de Medicina, Universidad Nacional de Colombia, Bogotá D.C., 111311 Colombia; 2 Institut Curie, PSL Research University, CNRS, UMR3244, Telomeres & Cancer lab, 75005 Paris, France; 3 Sorbonne Universités, UPMC Univ Paris 06, CNRS, UMR3244, 75005 Paris, France

**Keywords:** Senescence, IL6, IL8, Stemness, Breast cancer, Inflammation

## Abstract

**Background:**

There is compelling evidence associating senescent cells with the malignant progression of tumours. Of all senescence-related mechanisms, the so-called senescence-associated secretory phenotype (SASP) has attracted much attention. Since the pro-inflammatory cytokines IL-6 and IL-8 are consistently present in the SASP, and secreted by highly aggressive breast cancer cell lines, we aimed at elucidating their role on the less aggressive breast cancer cell line MCF-7, which does not secret these cytokines.

**Methods:**

The MCF-7 cell line was treated with either senescence-conditioned medium (SCM), IL-6 or IL-8 and then evaluated for phenotypic (CD44 and CD24 by FACS) and functional changes associated with an EMT program (migration/invasion) and for the acquisition of stem cell properties: mammosphere-forming capacity, expression of reprogramming factors (by qRT-PCR) and multilineage differentiation potential. We also evaluated the role of IL6 and IL8 in the cytokine-secreting, highly tumorigenic cell line MDA-MB-231.

**Results:**

Our results show that treatment of MCF-7 cells with IL6 and IL8, alone or together, induced the appearance of cells with fibroblastoid morphology, increased CD44 expression and migration, self-renewal and multilineage differentiation capacity, all characteristics compatible with an EMT program and stemness. These changes closely resembled those induced by a SCM. Interestingly, SCM treatments further increased IL6 and IL8 secretion by MCF-7 cells, thus suggesting the participation of an autocrine loop. Indeed, neutralizing antibodies against IL6 and IL8 reversed the effects of SCM on MCF-7, pinpointing these cytokines as major mediators of EMT and stemness-related effects associated with the senescent microenvironment. Additionally, prolonged exposure of MCF cells to IL6 or IL8 induced the appearance of senescent cells, suggesting a mechanism by which senescence and inflammation are reinforced favouring the acquisition of EMT and stem-like features at the population level, thus increasing tumour aggressiveness. Strikingly, our results also show that both IL6 and IL8 are important to maintain aggressive traits in MDA-MB-231 cells, a highly tumorigenic cell line, which appears to be devoid of stemness-related features.

**Conclusions:**

This study demonstrates that, similar to what is observed with a senescent microenvironment, purified IL6 and IL8 induce a self- and cross-reinforced senescence/inflammatory milieu responsible for the emergence of epithelial plasticity and stemness features, thus conferring more aggressive phenotypes to a luminal breast cancer cell line. On the other hand, the basal-like MDA-MB-231 cells, whose aggressiveness-related features depend on IL6 and IL8 secretion, almost completely lack mammosphere formation and differentiation capacities, suggesting that tumour aggressiveness is not always related to stemness.

**Electronic supplementary material:**

The online version of this article (doi:10.1186/s12964-017-0172-3) contains supplementary material, which is available to authorized users.

## Background

Cellular senescence has been considered as a powerful tumour suppressive mechanism [1, 2]. Nevertheless, early work has shown that cancer cells evade this mechanism in different ways [3–7]. Furthermore, senescent cells when co-transplanted with fully malignant cancer cells clearly increase cell growth and rate of tumour formation in xenotransplantation models [8–11]. There is also compelling evidence on the impact of senescent cells on the evolution of premalignant stages of tumour development [12, 13] and various types of premalignant cells are induced to proliferate and to form tumours in the presence of senescent fibroblasts [10, 14, 15]. The accumulated evidence indicate that a tumour-permissive microenvironment is induced by senescence-dependent mechanisms, either directly through cell-cell contacts or the secretion of tumour growth factors, or indirectly by modifying the environment in which tumour cells evolve.

Of all senescence-related mechanisms, the so-called senescence-associated secretory phenotype (SASP) has attracted much attention. Indeed, senescent cells secrete a wide assortment of soluble molecules [16], including inflammatory cytokines, chemokines, growth factors, and proteases believed to facilitate tumour growth. In vivo, SASP effects are thought to be much more complex because of its impact on immune, inflammatory and other stromal cells. SASP composition varies depending on cell and tissue of origin and the triggers involved [17]. Nevertheless, the inflammatory cytokines IL6 and IL8 are consistently present and known to be responsible for the maintenance and propagation of the SASP response in the microenvironment [16, 18]. Elevated serum levels of both cytokines have been used independently as prognostic markers for breast cancer [19–22]. Their effect has been principally and individually studied in basal/mesenchymal subtypes of breast cancer, since these cells secrete constitutively high amount of both cytokines [23–25]. In these models, IL6 has been shown to play a central role in the dynamic equilibrium between cancer stem cells (CSC) and non-CSC, and in the maintenance and enrichment of normal and malignant mammospheres [26–29]. Interestingly, it has been recently shown, in models of advances breast cancers HER2+, that IL6 was expressed mainly by senescent cells [30]. On the other hand, IL8 has been studied not only for its role as a chemokine in cancer invasion and metastasis [31, 32] but also for its ability to induce CSC activity in HER2+ breast cancer cells [33] and mammosphere formation in several cell lines of different origins [34]. Both cytokines are also essential for cell growth and anchorage-independent growth and colony formation in tumour cells from the so-called triple negative breast cancers (TNBC) [35]. Additionally, both cytokines play an essential role in mediating oncogenic-induced senescence acting in an autocrine and cell-autonomous fashion [36, 37].

These findings and others have revealed a close relationship between senescence and inflammation [17], and the relevance of the chronicity of both phenomena in tumour progression. For instance, prolonged SASP exposure can enhance tumour progression [10, 14] and chronic inflammation can increase cancer risk and act as a tumour-promoting stimulus [38]. Additionally, the decreased recurrence and mortality observed not only in colorectal cancer [39] but also in breast and pancreas cancer after surgical removal of the primary tumour and the use of the NSAIDs, suggest a possible common mechanism of action of the inflammatory mediators in tumour development [40, 41].

Recently, we have shown that post-crisis, pre-malignant HEK cells have the potential to become fully tumorigenic exclusively in the presence of a senescent microenvironment [15], and by doing so they acquire enhanced stem-like cell properties, autonomous tumorigenic potential and epithelial plasticity. Additionally, conditioned media from senescent fibroblasts (SCM), highly enriched in IL6 and IL8, strongly influenced the cell plasticity of pre- and fully malignant cells [15]. Nevertheless, SCM-induced spheres from CIN+ HEK cells did not form tumours, suggesting, that in this particular setting, the prolonged exposure to SASP is necessary for maintaining the senescence/inflammatory responses that confers tumorigenic properties.

Since luminal A breast cancer cell lines do not express IL6 or IL8 [25, 42, 43], the relevance of these cytokines in CSC function has been poorly studied. Nevertheless, they could play a role in luminal A breast cancer development and progression. The understanding of the contribution of these specific cytokines in maintaining a tumour prone milieu capable of inducing specific properties of tumour cells would be extremely relevant for novel therapeutic interventions. In the present work, we have studied the specific contribution of the pro-inflammatory cytokines IL6 and IL8 in the acquisition of a tumorigenic phenotype using the well-characterized luminal breast cancer cell line MCF-7, which does not express IL6 or IL8 constitutively [25, 42, 43]. This cell line has been classified as senescent-cell progenitor subtype with abilities to differentiate into luminal and myoepithelial cell types, and showing lower and heterogeneous expression of the senescence-associated beta-galactosidase (SA-βGal). This is in clear contrast with other cell lines, including the MDA-MB-231 basal breast cancer subtype, which expresses high IL6 and IL8 levels and are categorized as immortalized-cell progenitor subtype with limited differentiation potential and negative for SA-βGal [44].

## Methods

### Breast cancer cell lines and culture conditions

MCF-7 and MDA-MB-231 cells were obtained from the ATCC and were maintained under standard culture conditions in a humidified 5% CO2 atmosphere at 37 °C. MCF-7 cells were grown in minimum essential medium, MEMα (Invitrogen) supplemented with 10% heat inactivated fetal bovine serum (FBS), nonessential amino acids and sodium pyruvate. MDA-MB-231 cells were cultured in Dulbecco’s modified Eagle’s medium DMEM (Invitrogen) supplemented with 10% heat inactivated FBS.

### MCF-7 treatment with senescent-conditioned medium (SCM)

SCM was prepared by culturing senescent (PD65) foreskin fibroblasts HCA2 to 80% confluence, then washed twice with PBS 1× and serum starved for 48 h in Iscove’s modified Dulbecco’s media + glutamax culture media (Invitrogen) without FBS. SCM was collected, centrifuged, filtered and its concentration normalized to the number of cells used. MCF-7 cells were treated with either normal or SCM for 5 days with refeeding every two days.

### Senescence-associated β-galactosidase (SA-βGAL) assay

Senescence-associated β-galactosidase (SA-βGAL) activity was evaluated in senescent- and young fibroblasts after 2 days of culture by using the cellular senescence assay kit (KAA002, Millipore). SA-βGAL activity was also determined in MCF-7 cells treated for 5 or 10 days with high (50 ng/ml) or low (0,5 ng/ml) cytokine concentration or SCM. Briefly, cells were seeded in triplicates in 24-well plates, washed twice with PBS 1×, and then fixed with formalin for 5 min at room temperature. Next, cells were washed twice with PBS 1× and incubated overnight at 37 °C with β-gal substrate in an acidic buffer (pH 6.0) and examined with an inverted microscope and photographed (NIKON TS100). The development of a perinuclear blue color was indicative of senescent cells.

### MCF-7 treatment with human recombinant cytokines or neutralizing antibodies

Adherent MCF-7 cells were grown to 20% confluence in complete medium, washed twice in PBS 1× before the addition of 50 ng/ml of human recombinant IL8 (PHC0084, Invitrogen), IL6 (PHC0065, Invitrogen) or the mixture (IL6 + IL8) in 0,5% FBS MEMα medium. For neutralization experiments, MCF-7 cells were incubated with SCM containing 1 μg/ml of human CXCL8/IL-8 antibody (AF-208-NA, R&D SYSTEMS), IL6 antibody (MAM206, R&D SYSTEMS) or the mixture (anti-IL6 + anti IL8). Cells cultured in MEMα supplemented with 0,5% FBS or SCM were used as a control. Cells were cultured for 5 days and cytokines or antibodies were added every two days.

### Cell proliferation determination

SCM-or cytokine-treated MCF-7 cells or control were seeded in triplicates into 16-mm-diameter wells at a density of 5000 cells per well in 0,5% FBS MEMα medium and allowed to attach for 24 h. At daily intervals and for 5 consecutive days, cells were harvested from the monolayer after trypsinization and counted using a Neubauer chamber.

### Cell migration and invasion assays

SCM-or cytokine-treated MCF-7 cells or control were grown to 80–90% confluence then serum starved overnight before setting up the experiment. Cells were washed twice with PBS 1×, harvested after trypsinization and collected and suspended in starvation medium. For “wound healing” assay, cells were seeded in triplicates, grown to confluence and scratched with a p10 pipette tip making a straight scratch, simulating a wound. Images were captured at 0, 6 and 12 h. At least 10 images at each time point were used for analysis and the percentage of invaded area was estimated by using Image J program. For transwell migration assays, filters (8.0 μm pore size) and 24-well transwell chambers (BD Biosciences) were used. Chambers were rinsed with culture medium without serum 1 h before the assay. The cells were plated in triplicates in the upper wells at a density of 1 × 10^5^ per well in 0,1 ml; the lower chambers were set in the following conditions: complete medium + 50 ng/mL hrIL8 or complete medium (20% FBS). Cells were allowed to migrate for a period of 48 h at 37 °C and 5% CO2 atmosphere, after which the experiment was stopped by wiping the cells from the well with a cotton swab and fixed with methanol 15 min and then stained with 0,5% Crystal violet in water for 15 min. A total of 10 images were taken for quantification using an inverted microscope. The invasion assay was identical to the above migration assay except that filters were coated with 100 μL of matrigel (BD Bioscience), diluted one-third in media without serum. The experiment was stopped after 48 h as described above.

### Cell adhesion assay

Fibronectin at 20 μg/ml (Invitrogen) or Collagen at 40 μg/ml (Sigma) was added to 96-well plates and incubated for 2 h at 37 °C and 5% CO2 atmosphere; then the solution was removed and the wells were washed with PBS 1× and incubated during 1 h with the blocking solution (0,5% of BSA in medium). 4 × 10^4^ cells were added to each well in triplicates. Plates were then cultured for 40 min at 37 °C and 5% CO2 atmosphere, after which the medium was removed. The non-adherent cells were washed twice with PBS 1× and the remaining cells were stained with Crystal violet as described above. The adherent cells were counted using an inverted microscope.

### Flow cytometry analysis

Cells (10^6^) were harvested after trypsinization, washed twice (for cell surface markers expression) or fixed with ethanol (for Ki-67 staining) and suspended in 500 μl PBS 1X containing 0,5% bovine serum albumin. Cells were stained with FITC-conjugated mouse anti-human CD44 and R-Phycoerythrin-conjugated mouse anti-human CD24 (both from Invitrogen). Cells incubated without antibody were used as a blank. For Ki-67 determination an allophycocyanin-conjugated mouse anti-human Ki-67 (Biolegend) and its respective isotype control APC Mouse IgG1, κ isotype Ctrl (FC) were used. The different antibodies were incubated for 30 min and washed to remove the excess of antibodies. The cytometric analysis was carried out using a fluorescence-activated cell sorting (FACS) Aria-II flow cytometer (BD Bioscience). The Flow Jo software was used for data acquisition and analysis, respectively, using measurements from 10,000 cells in each experiment.

### Human inflammatory cytokine assay

The amount of pro-inflammatory cytokines (IL-1β, TNF-α, IL-12-p70, Il-6, IL-8, IL-10) present in cell supernatants from senescent and young fibroblasts or SCM-treated or untreated MCF-7 was determined using a human inflammatory cytokine kit (BD™ Cytometric Bead Array (CBA) following instructions of the manufacturer. A FACScan flow cytometer (BD) was used to analyse samples.

### Quantitative reverse transcription polymerase chain reaction (qRT-PCR)

Cells were collected by centrifugation, and RNA was extracted using the Trizol/chloroform method (Invitrogen) according to manufacturer’s instructions. The concentration of total RNA was measured by a ND-1000 NanoDrop spectrophotometer and one microgram of RNA was treated with DNAse I (Invitrogen) and used for the reverse transcription using the high capacity cDNA reverse transcription kit (Applied Biosystems). The resulting cDNA was diluted 1:4 and assessed by qRT-PCR using Power Syber Green Master Mix (Applied Biosystems). The volume of each reaction was 25 μL. Measurements were done in a 7500 Real Time PCR system. For each sample, qRT-PCR reactions were done in triplicate, and the entire analysis was done twice independently. Ct-values for each sample and the data were exported to Microsoft Excel for further analysis. The average Ct-value for the endogenous control (GADPH) was calculated for each sample. To calculate the relative expression of the gene of interest the delta-delta Ct-method was used [45]. The sequence of the primers used is provided in the Additional file 1: Table S1.

### Mammosphere-forming assay

For the mammosphere-forming assay, cultured MCF-7 and MDA-MB-231 cells at a density of 5 × 10^3^ cells/mL per well were suspended and seeded in triplicates into ultralow attachment plates (MW6) (Nalge Nunc Interanational), in serum free DMEM/F12 (1:1) medium supplemented with 20 ng/mL basic-fibroblast growth factor (b-FGF, Gibco; Ref. PHG0266), 20 ng/mL epidermal growth factor (EGF, Gibco; Ref. PHG0315), ITS (insulin + transferrin + selenium, Sigma), B27 supplement (GIBCO) and 1% methylcellulose. Fresh medium was added to each well every two days (without removing the old medium). Cells were grown in these conditions as non-adherent spherical clusters of cells during 3–9 days after which the spheres (>100 μm) were counted. Media without growth factors was used as a control (without induction). Second and third-generation mammosphere were formed by collecting mammosphere every five days by gentle centrifugation and dissociated to single cell suspensions by incubation in a 0,25% trypsin-EDTA solution for about 5–10 min at 37 °C and by mechanical dissociation by pipetting up and down 10–20 times. The single cells suspensions were washed, counted and plated at the initial density in the defined media describe above.

### Differentiation assays to mesenchymal lineages

For adipogenic and osteogenic differentiation assays, 2 × 10^4^ cells were seeded in triplicates in a 24-well format plate and cultured in complete medium. 24 h later, the medium was replaced with the respective induction medium. For adipogenic differentiation we used incomplete medium MEMα (Sigma–Aldrich) supplemented with 10% FBS, 1 mM dexamethasone (Sigma), 0,5 mM isobutylmethylxanthine (Sigma), 200 μM indomethacin (Sigma–Aldrich) and 10 μg/ml insulin (Sigma–Aldrich). After 3 days, maintenance medium containing MEMα, 10% FBS and 10 μg/ml insulin, was added to the cells. Three cycles of induction and maintenance were completed. After 10 days, cells were washed twice with PBS 1×, followed by fixation with formalin for 30 min (Sigma–Aldrich) and stained with Oil Red- O solution for 1 h (Sigma–Aldrich). The number of Oil Red-O positive cells was determined with an inverted microscope [46, 47]. Osteogenic differentiation was induced with incomplete medium MEMα, supplemented with 10% FBS, 100 nM dexamethasone, 0,2 mM ascorbic-2-phosphate (Sigma–Aldrich) and 10 mM β-glycerophosphate (Sigma–Aldrich). Medium was changed every 3–4 days. After 10 days, cells were assessed for alkaline phosphatase activity (APL activity) using an APL staining kit (Chemicon International, SCR004) following the manufacturer’s instructions; cells were examined with an inverted microscope [48]. For chondrogenic differentiation, 5 × 10^4^ cells were plated in a 24-well plate and cultured in chondrogenic induction medium, containing MEMα and 10 ng/ml TGFβ-1 (Sigma–Aldrich). The medium was changed three times a week. After 10 days, the cells were washed twice with PBS 1×, followed by fixation with formalin for 5 min and stained with 0,1% Safranin O for 3 min (Sigma–Aldrich). Cells were observed with an inverted microscope [49].

### BrdU proliferation assay

MCF-7 cells at 30-50% confluence were cultured overnight in 25 cm^2^ flasks. After 24 h of serum starvation, cells were treated with cytokines for 12 h or with SCM during 5, 7 and 10 days. After treatment, cells were labelled with 30 μM BrdU (Eurobio) for 25 min, washed and permeabilized with 100% ethanol. After treatment with pepsin and HCl 2 N to denature the DNA, samples were incubated with primary anti-BrdU and secondary anti-BrdU-FITC antibodies (Invitrogen). Samples were analyzed by flow cytometry.

### Transfection of MDA-MB-231

MDA-MB-231 cells were transfected with pSuperRetropuro-IL-6 shRNA 1 plasmid (Addgene) or endoribonuclease-prepared small interfering (esi)RNAs (Sigma EHU 048321) using an Amaxa Nucleofection system (Amaxa Biosystems, Gaithersburg, MD) or Lipofectamine 2000 (Life Technologies, Carlsbad, CA) according to the manufacturers’ instructions. PSuperRetroPuro empty plasmid and esiFLUC (Firefly luciferase) were used as a control.

### Statistical analysis

Comparisons for gene expression levels, cell growth, sphere-forming, adhesion, migration and invasion capacities were analyzed with GraphPad Prims v5 software using non-parametric two-tailed *t* test (Mann–Whitney) and two way ANOVA with Bonferroni post-test. Significant *p* value was considered as * <0.05, ** <0.01 or *** <0.001.

## Results

### SCM induces an EMT-like program in MCF-7 cells

**Fig. 1:**
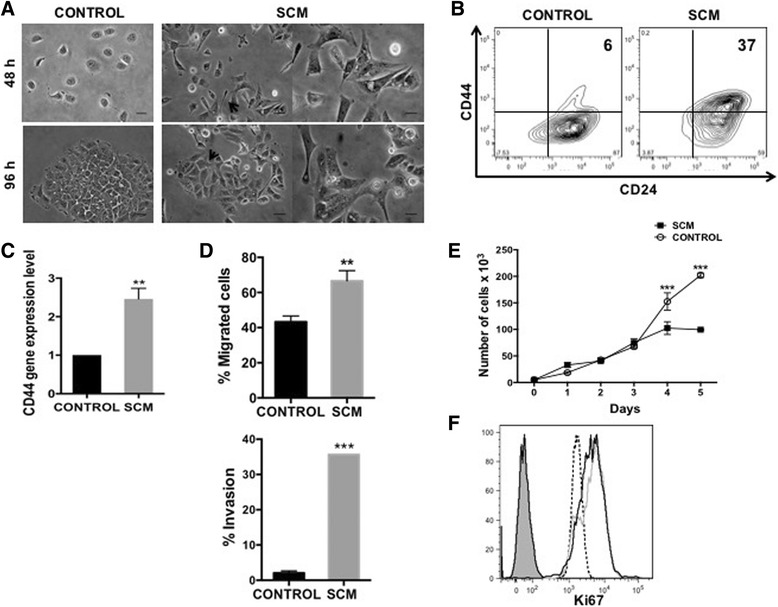
The SCM induces an EMT-like program in MCF-7 cells. **a** MCF-7 cells were cultured in media with 0,5% FBS (control) or SCM. The morphological evaluations were done at 48 and 96 h after SCM treatment. Representative images are shown. *Arrowhead* indicates cells with fibroblastoid morphology. Scale bar, 10 μm. **b** Representative plot of surface marker expression determined by flow cytometry (FACS) with CD44-FITC (mesenchymal marker) and CD24-PE (epithelial marker) monoclonal antibodies in MCF-7 cells stimulated with SCM during 5 days (*n* = 2). **c** CD44 expression determination by qRT-PCR; the values were normalized to GADPH and relative to control cells. Error bars represent SEM (*n* = 2). **d** Transwell migration (*upper*) and matrigel invasion (bottom) assays performed in MCF-7 cells control and treated with SCM by using 20% FBS as a quimioattractant in the lower compartment. The histograms show the number of cells present in the lower compartment as a measure of the ability of this cells to migrated (*upper*) and the number of cells present on the bottom surface of filters from at least 15 images as a measure of the ability to invade (*bottom*). Error bars indicate SEM. (**p < 0.01) (*n* = 2). **e** Growth kinetics of MCF-7 cells upon treatments with SCM. Equal numbers of cells were seeded by triplicate and cells were counted at the indicated time points. Error bars represent SEM. (***p < 0.001) (*n* = 2). **f** Representative FACS histograms showing the expression of Ki-67 in control and SCM-treated cells (*black* and *grey* histogram, respectively). Filled histogram corresponds to blanc and dotted histogram to isotype control

Determining the particular role of specific components of the SASP responsible for conferring special competencies to pre- or fully tumorigenic cells is of great relevance in tumour biology and therapeutics. In this regard, it is interesting to point out that a senescent microenvironment is able both to uncover the tumorigenic potential of premalignant cells and to influence the metastable differentiation of pre- and fully tumorigenic cells [15]. Although the SASP composition may vary the pro-inflammatory cytokines IL6 and IL8 are consistently expressed by senescent cells [18]. We sought to investigate if a senescence-associated inflammatory milieu could also influence the tumorigenic and differentiation potential in a luminal A breast cancer cell model. We therefore studied the MCF-7 human cell line, characterized as an estrogen and progesterone receptor positive (ER+/PR+) and Her-2/neu negative (Her2-) cell line, exhibiting low tumorigenic capacity, and importantly, expressing low or no detectable levels of endogenous IL6 or IL8 [25, 42, 43]. Exposure of MCF-7 cells to SCM from HCA2 senescent fibroblast (Additional file 2: Figure S1A) induced a well-defined morphological change with the loss of cobblestone-like appearance and the presence of cells that adopted an elongated, spindle-like shape more typical of fibroblastoid cells (Fig. 1a). Interestingly, SCM-treated MCF-7 cells showed increased expression of the mesenchymal and stem cell-associated marker CD44 [50, 51], as evaluated by FACS (Fig. 1b) and qRT-PCR (Fig. 1c), in contrast to control MCF-7 cells, which exhibited an epithelial-like morphology (Fig. 1a) and displayed a very low proportion of CD44+ cells (Fig. 1b). These changes were accompanied by an increase in the directed migration towards FBS and matrigel invasion capacity of MCF-7 (Fig. 1d, upper and lower panel, respectively). On the other hand, the SCM did not induce cell growth in MCF-7 cells in the first three days of culture (Fig. 1e). After 4–5 days of incubation with SCM, MCF-7 cells showed less cell numbers than control cells. Nevertheless, Ki-67 expression was determined at day 5^th^ of treatment and showed no difference between control and SCM-treated cells (Fig. 1f). Additionally, epithelial and mesenchymal markers were measured after SCM addition. MCF-7 cells showed increased expression of vimentin, ZEB-1, SNAIL-1 and SNAIL-2/Slug (without losing E-cadherin or TJP-1 expression) (Additional file 2: Figure S1C), and of the cell reprogramming factors OCT-4 and KLF-4 (Additional file 2: Figure S1D). These results give suggestive evidence that MCF-7 cells undertake an epithelial-mesenchymal transition (EMT) program when in contact with an SCM.

### IL6 and IL8 treatments reproduce the morphological, phenotypic and functional changes induced by the SCM

Cellular senescence is accompanied by a striking increase in the secreted levels of more than 40 soluble factors, the SASP [52]. We were particularly interested in exploring the contribution of senescence-associated pro-inflammatory cytokines in the observed changes after SCM treatment. Therefore we quantified the amount of some pro-inflammatory cytokines (IL1β, TNFα, IL12-p70, IL6, IL8, IL10) by CBA and found that the SCM was highly enriched in IL6 and IL8 (Additional file 2: Figure S1B), two pleiotropic pro-inflammatory cytokines that have been implicated in cancer progression [32, 43]. Conditioned media from young fibroblasts do not produce these or other of the pro-inflammatory cytokines here tested (Additional file 2: Figure S1B).

**Fig. 2:**
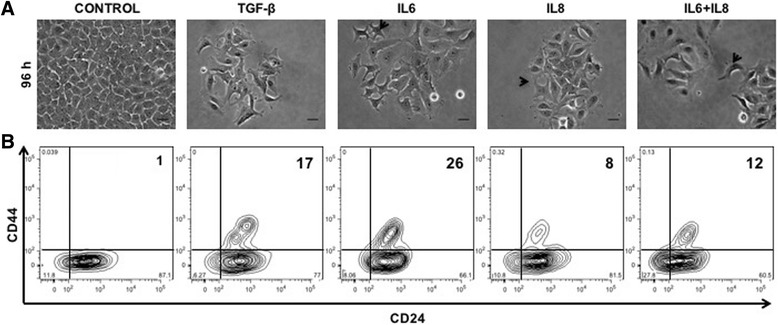
The treatment with IL6 and IL8 induced morphological and phenotypic changes associated with an EMT process. **a** MCF-7 cells stimulated with 50 ng/ml of IL6, IL8 or IL6 + IL8 cytokines. 5 ng/ml of TGF-β was used as a positive control and MCF-7 cultured in MEM-α medium supplemented with 0,5% FBS was used as a negative control. The evaluation was done after 96 h of cytokine treatment. Representative images are shown. *Arrowhead* indicates cells with fibroblastoid morphology. Scale bar, 10 μm. **b** Representative plot of fluorescence intensity of the EMT surface markers was determined by FACS using a CD44-FITC and a CD24-PE monoclonal antibody in MCF-7 cells stimulated for 5 days with cytokines (*n* = 2)

**Fig. 3:**
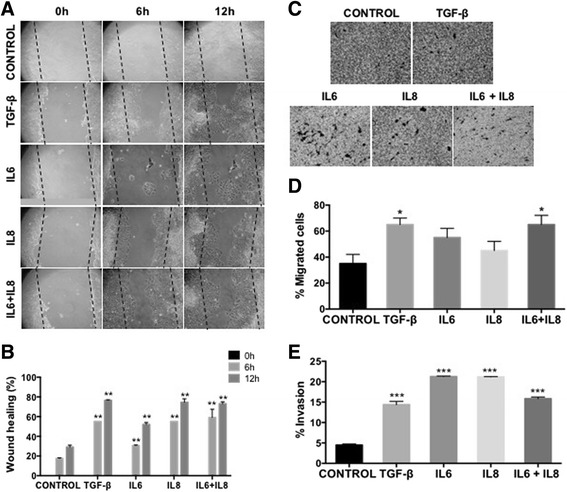
The treatment with IL6 and IL8 increases the migration capacity of MCF-7 cells. **a** Wound healing assays performed in MCF-7 cells treated with cytokines. Representative micrographs (10×) were taken at 0, 6 and 12 h showing an increased migration capacity of treated MCF-7 cells. *Black dotted line* indicated the area of the wound. **b** Quantification of the area of wound healing assay from at least 10 images by using the image J program. Error bars indicate SEM. (**p < 0.01). **c** Transwell migration assay towards 20% FBS performed in cytokine-treated and control MCF-7 cells. Representative micrographs (20×) were taken from the membrane filter (bottom surface of filters) stained with Crystal violet. **d** Quantification of transwell migration assay by counting the number of cells present in the lower compartment. Error bars indicate SEM. (*p < 0.05). **e** Transwell matrigel invasion assay towards 20% FBS performed in cytokine-treated and control MCF-7 cells. The histograms show the number of cells present on the bottom surface of filters from at least 15 images. Error bars indicate SEM. (***p < 0.001) (*n* = 2)

We therefore studied the effects of human recombinant IL6 and IL8 individually or together on the MCF-7 cell line. As a positive control of the EMT program, we used the well-known EMT-inducer TGF-β [53–55]. After 5 days of exposure to IL6 or IL8 or both, MCF-7 cells adopted a more fibroblastoid morphology with a concomitant increase in CD44 expression (Fig. 2a-b), similar to TGF-β and to the above-described effects of the SCM. The stimulation on CD44 expression was visible either by FACS (Fig. 2b) or by qRT-PCR (Additional file 3: Figure S2A), and it was neither additive nor synergistic when both cytokines were used (Fig. 2b and Additional file 3: Figure S2A). As observed with the SCM, cytokine-treated MCF-7 cells were more motile, with cells spontaneously migrating more than control cells or having higher migration towards FBS, as assessed by the “wound-healing” (Fig. 3a-b) or the transwell migration assays (Fig. 3c-d). BrdU labelling of DNA showed that cytokine-induced “wound healing” was not due to MCF-7 cell proliferation; no changes in the cell populations in the Go/G1, S and G2/M phases of the cell cycle were observed (Additional file 3: Figure S2B). An important increase in Matrigel invasion capacity was also evident after cytokine treatments (Fig. 3e). Although there were statistically significant differences in migration and invasion capacity between the control and the different cytokine treatments, differences between treatments were minimal. Here again, there were no major differences in cell proliferation rates in MCF-7 cells treated with cytokines and control, similar to what we observed with SCM-treated MCF-7 cells (Additional file 4: Figure S3A). Despite slight variations, it appears that the different cytokine treatments (IL6, IL8 or both) induced morphological, molecular and functional changes compatible with an EMT process and that they closely resemble those observed with the SCM.

**Fig. 4:**
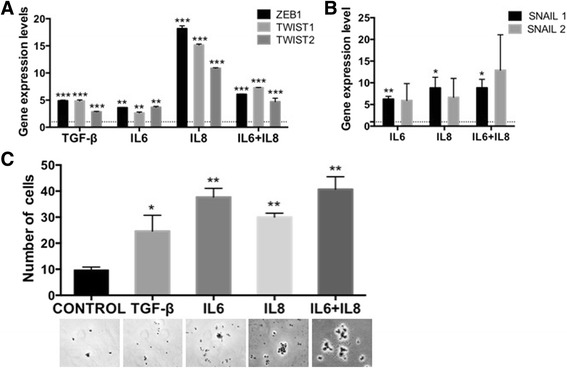
The treatment with IL6 and IL8 induced gene expression and functional changes associated with an EMT program. Gene expression levels of EMT-associated transcription factors Zeb1, Twist 1 and Twist 2 (**a**) and Snail 1 and Snail2/Slug (**b**) in MCF-7 cells stimulated with cytokines as indicated and determined by qRT–PCR. The values were normalized to GADPH and relative to control cells (*dotted lines*). Error bars represent SEM. (*p < 0.05; **p < 0.01; ***p < 0.001) (*n* = 2). **c** Adhesion of MCF-7 cells to immobilized-fibronectin after cytokine treatment. Representative micrographs (20×) taken from the plates stained with Crystal violet. The number of adherent cells was counted. Error bars indicate SEM. (*p < 0.05; ** *p* < 0.01)

To further characterize this response, we proceeded to evaluate other EMT markers, including E-cadherin, cytokeratin 18, the tight junction protein 1 (TJP-1) and vimentin. There was not a well-defined pattern of changes in relation with these markers (Additional file 4: Figure S3B), as it was the case for SCM. Nevertheless, analyses by qRT-PCR of the EMT-associated transcription factors (TFs), ZEB1, TWIST-1 and TWIST-2 consistently showed a gene expression pattern totally consistent with an EMT (Fig. 4a). While the levels of ZEB1, TWIST-1 and TWIST-2 augmented in a similar way (about three times) in response to IL6, the mixture of IL6 and IL8, and TGF-β, they reached very high levels upon IL8 treatment (between 10 and 15 times). The upstream regulators SNAIL-1 and SNAIL-2/Slug were also evaluated and only SNAIL-1 consistently showed increased gene expression (Fig. 4b). In addition, cytokine-treated MCF-7 cells showed more ability to adhere to fibronectin, a process that has been associated with an EMT event [56] (Fig. 4c). Altogether, these results are clearly indicative of an EMT program induced by the cytokines IL6 and IL8, quite similar although not identical to the one induced by the SCM.

### Cytokines-treated MCF-7 cells exhibit stem-like cell properties

**Fig. 5:**
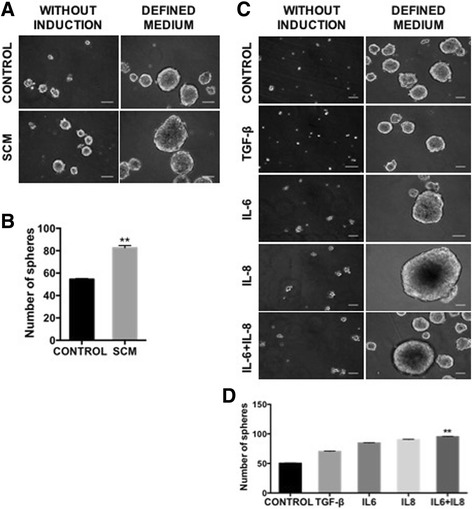
MCF-7 cells treated with IL6 and IL8 exhibit self-renewal properties. **a** Sphere formation assay in the presence of defined medium (EGF and FGF) in MCF-7 cells that were previously treated or not (Control) with SCM (**a** and **b**) or with cytokines (**c** and **d**) during 5 days. Representative micrographs (10×) show sphere formation after 8 days of induction. Scale bar, 100 μm. The total number of spheres per well larger than 100 μm was counted at day 8. Error bars indicate SEM. (**p < 0.01) (*n* = 2)

It has been previously shown that the EMT program is frequently accompanied by the emergence of cells with stem-like properties [50, 57–59]. Therefore, we studied the SCM effect on MCF-7’s stemness potential by first evaluating the sphere-forming capacity [60]. MCF-7 cells in defined medium displayed limited capacity to form mammospheres (<100 μm). However, when MCF-7 cells were pre-treated with SCM, their capacity to form spheres increased (by >50% compared to control) (Fig. 5a-b). Interestingly, spheres formed in the latter condition were, in some cases, bigger than 500 μm. Next, we evaluated the sphere-forming capacity after incubation with either IL6, IL8 or both. These pre-treatments, which by themselves did not efficiently induce sphere formation, increased the number (by almost 50%) and the size (by at least 5 times) of mammospheres when compared to control cells in defined medium alone, or to the TGF-β-treated cells (Fig. 5c-d). This increase in sphere size, seen with SCM and most particularly with IL6 and IL8, suggested some degree of mammosphere fusion. In order to assess more precisely the effect of cytokine pre-treatment on sphere formation potential we performed early and daily evaluations. As illustrated in Additional file 5: Figure S4A, both IL6 and IL8 treatments induced 10 times more spheres at day 3 and 15 times more spheres at day 4 than the control (no pre-treatment), respectively. Although some fusion events were detected at early time points (Additional file 5: Figure S4B), the data support the idea that IL6 and IL8 are able to very efficiently induce mammosphere formation. Self-renewal capacity was confirmed in experiments where serial passages allowed sphere formation for at least three generations (Additional file 5: Figure S4C).

**Fig. 6:**
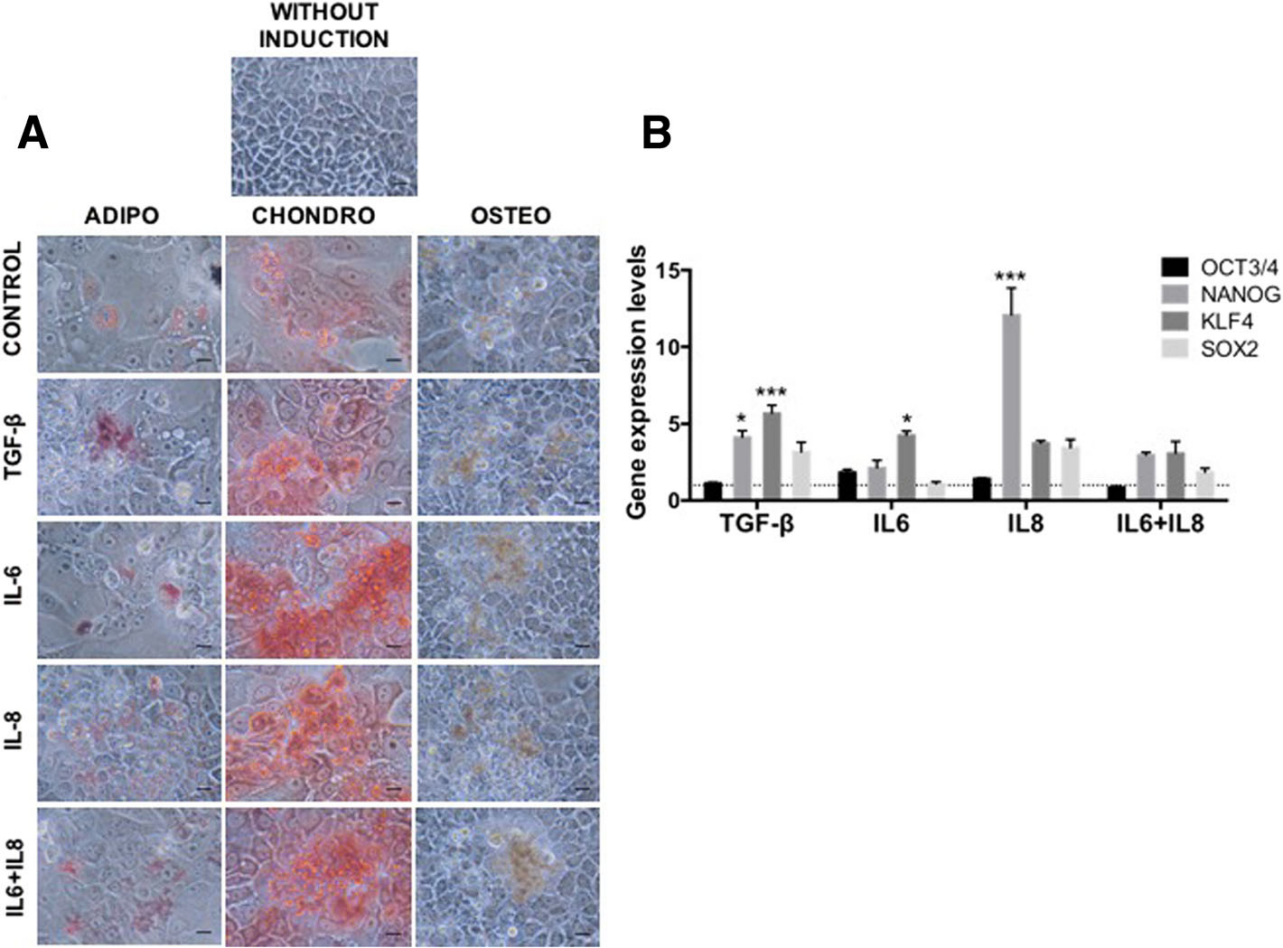
MCF-7 cells treated with IL6 and IL8 exhibit differentiation potential characteristics and express reprogramming factors. **a** Differentiation to mesenchymal cell lineages was induced by using specific induction media as described in Materials and Methods and evaluated after 10 days of induction. Osteoblasts (ALP activity), adipocytes (oil red-O) and chondrocytes (Safranin O) differentiation was determined with specific staining. Representative images are shown. Scale bar, 10 μm. **b** Gene expression levels of reprogramming-associated transcription factors in MCF-7 cells stimulated with cytokines as indicated and determined by qRT–PCR. The values were normalized to GADPH and relative to control cells (*dotted lines*). Error bars represent SEM (*p < 0.05) (*n* = 2)

To further explore the impact of cytokines on the emergence of stemness properties of tumor cells, we determined the multilineage differentiation capacity of MCF-7 cells. We found that after treatment with either IL6, IL8 or both differentiation induction into mesenchymal lineages occurred remarkably fast (10 days compared to the 2–3 weeks needed for the differentiation of bone-marrow mesenchymal stem cells [61, 62]), and very efficiently (as judged by the intense and more homogenous staining in the culture dish) (Fig. 6a). Cytokine-untreated MCF-7 cells also exhibit a slight differentiation capacity (Fig. 6a, control), suggesting a pre-existing condition that could be strengthened by a senescence-associated pro-inflammatory microenvironment. A similar observation can be made regarding the assays described above in which cytokine pre-treatments potentiate the formation of spheres (Fig. 5c).

Given that poorly differentiated tumours tend to have more stem properties with preferential overexpression of reprogramming TFs [63], we evaluated their gene expression level in response to cytokine treatments. Interestingly, IL8 treatment enhanced the expression of NANOG, KLF-4 and SOX2, whereas IL6 enhanced only the expression of KLF-4, similar to the TGF-β. Treatment with both IL6 and IL8 produced a slight increase in these TFs (Fig. 6b). OCT4 expression was not modified by cytokine treatment. As with EMT-TFs, IL8 gives a strong reprogramming TF response, especially NANOG.

**Fig. 7:**
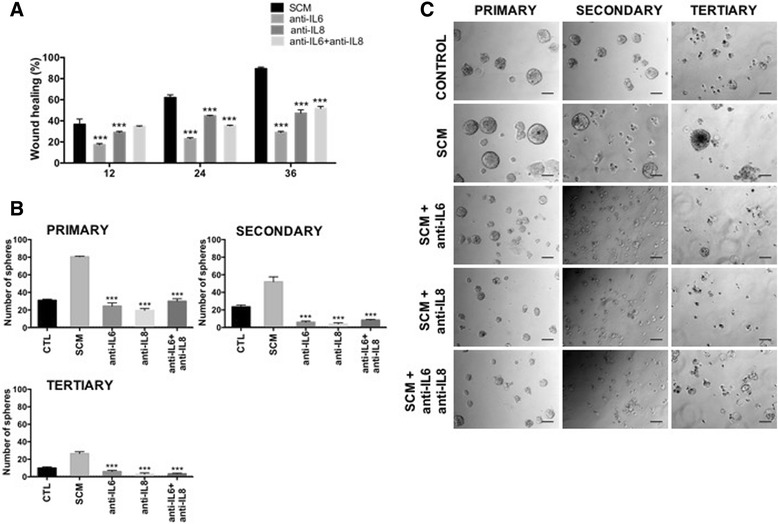
The pro-inflammatory cytokines IL6 and IL8 are required for the maintenance of the migration capacity and stem cell properties in MCF-7 cells. MCF-7 cells were incubated with SCM with or without 1 μg/ml of neutralizing monoclonal antibodies against IL6 (Ref: MAB206, R&D systems) and IL8 (Ref: AF-208-NA, R&D systems). **a** Wound healing assays showing a decrease in the migration capacity of treated MCF-7 cells with the neutralizing antibodies. The histograms show the quantification of the area of wound healing from at least 10 images taken at 12, 24 and 36 h, using the image J program. Error bars indicate SEM. (***p < 0.001). **b** Sphere formation assay in the presence of defined medium (EGF and FGF) in MCF-7 cells that were previously treated or not (Control) with SCM alone or in the presence of neutralizing antibodies. The total number of primary, secondary and tertiary spheres per well larger than 100 μm was counted at day 5. Error bars indicate SEM. (***p < 0.001) (*n* = 2). **c** Representative micrographs (10×) showing primary, secondary and tertiary generations of mammospheres. Scale bar, 100 μm

The relevance of IL6 and IL8 in the acquisition of these important functional properties was further evaluated by incubation of SCM with neutralizing monoclonal antibodies against these cytokines (Additional file 6: Figure S5A). Inhibition of IL6 and IL8 by specific antibodies was around 50 to 65% as it was for MCF-7 cell migration, as evaluated by the wound-healing assay (Fig. 7a). Mammosphere formation capacity was also importantly reduced (60%) in the first generation and even more in subsequent generations (Fig. 7b and c). SNAIL-1, SNAIL-2/Slug and KLF4 expression was consistently reduced by antibodies treatment (Additional file 6: Figure S5B and C).

Overall, our results show that MCF-7 breast cancer cells in a senescence-associated pro-inflammatory milieu can acquire characteristics of more aggressive cells, i.e.,: a mesenchymal morphology with higher mobility and CD44 expression, thus resembling an EMT program, as well as acquisition of stem-like cell properties such as self-renewal and multi-differentiation potential. Importantly, IL-6 and IL-8, present in the SCM, play a major role in the acquisition of these properties.

### Senescence and inflammation are self- and cross-reinforcing

**Fig. 8:**
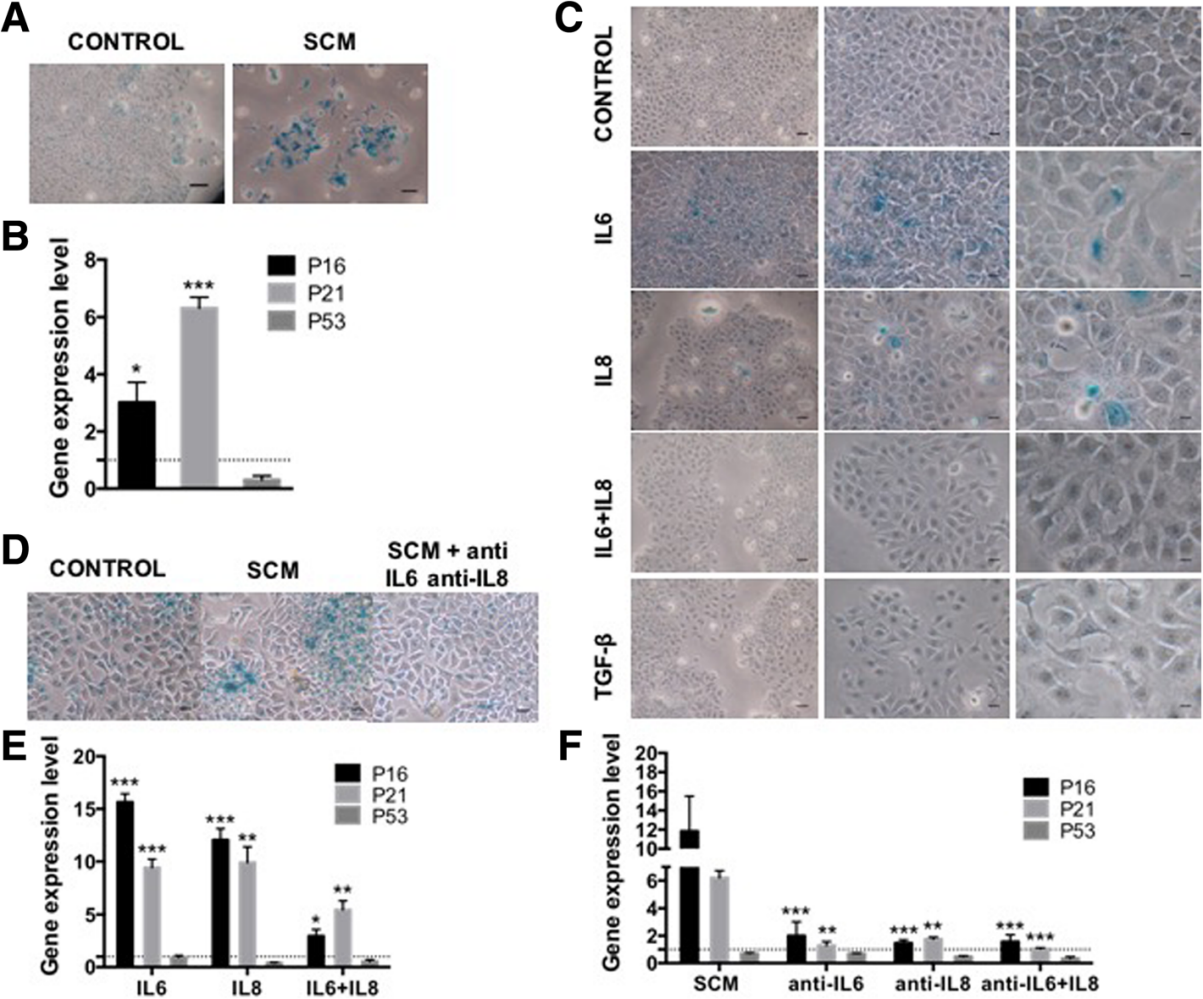
The treatment with IL6 and IL8 induces senescence in MCF-7 cells. **a** Representative images of MCF-7 cells treated with SCM during 10 days or (**c**) cytokines (50 ng/ml) during 5 days and stained for SA-β-GAL. Scale bar, 10 μm. **b** Gene expression profile of p16, p21 and p53 in MCF-7 cells stimulated with SCM or (**e**) cytokines, as indicated. The values were normalized to GADPH and relative to control cells (*dotted lines*). Error bars represent SEM. (*p < 0.05; **p < 0.01; ***p < 0.001) (*n* = 2). The incubation of SCM with neutralizing anti-IL6 and anti-IL8 antibodies suppresses SA-βGal activity (**d**) and reduce the expression of senescent markers P16, P21 and P53 (**f**) determined by qRT-PCR. Error bars represent SEM. (**p < 0.01; ***p < 0.001) (*n* = 2)

In the course of these experiments, we noticed that the MCF-7 cells looked slightly flattened after SCM treatment (Fig. 1a). We therefore proceeded to evaluate the senescent-associated β-galactosidase (SA-βGal) activity, which was negative after 5 days of exposure to SCM (Additional file 7: Figure S6A). Yet, incubation of MCF-7 cells with SCM induced a further increase in the expression of IL6 and IL8 (six times for IL8 and at least two times for IL6) (Additional file 7: Figure S6B, to be compared to Additional file 2: Figure S1B). This observation suggested the involvement of an autocrine mechanism that could reinforce the effect of the SCM. Remarkably, a prolonged (10 days) exposure to SCM induced the appearance of senescent cells as evaluated by SA-β activity (Fig. 8a) or p16 and p21 expression (Fig. 8b). A similar result was obtained by stimulating MCF-7 cells with either IL6 or IL8, but not with TGF-β or a combination of IL6 and IL8 (Fig. 8c). Incubation of SCM with anti-IL6 and anti-IL8 antibodies suppressed SA-βGal activity (Fig. 8d). IL6 and IL8 also induced a significant increase of p16 and p21 (Fig. 8e) that was abrogated by incubation with anti-IL6 or/and IL8 antibodies (Fig. 8f). These observations confirm that a senescence process is induced by the IL6 and IL8 present in the SCM. Also, exposure to lower (100 times less) IL6 or IL8 concentrations for 10 days induced positive SA-βGal activity in MCF-7 cells (Additional file 7: Figure S6C) as did a prolonged exposure to high TGF-β concentration. In the case of IL6 and IL8, it appears that prolonged exposure to lower cytokine concentrations is more effective in senescence induction than shorter stimulation with a high cytokine concentration. Furthermore, we observed that the proportion of senescence cells induced by SCM did not change after 48 h of contact with serum containing medium suggesting that the senescence state was irreversible (Additional file 7: Figure S6D).

These results suggest that a senescent-induced pro-inflammatory microenvironment triggers a cellular mechanism by which both senescence and inflammation are reinforced at the cell population level. This, in turn, should favour the appearance of cells bearing stem cell attributes (EMT-like program, enhanced cell migration, self-renewal and differentiation capacity) allowing them to migrate to new senescence/inflammatory niches. Indeed, we observed that MCF-7 cells incubated with SCM migrate more efficiently towards IL8 (Additional file 7: Figure S6E).

### IL6- and IL8-secreting MDA-MB-231 cells displayed an EMT-like phenotype but exhibit partial/incomplete stem-like cell capabilities

Taken together, these observations suggest that a senescence-induced inflammatory microenvironment could make a cancer cell adopt a more aggressive tumorigenic potential. Indeed, basal/mesenchymal breast cancers that constitutively express IL6 and IL8 have been shown to be more aggressive than luminal cancers [64–66]. To further evaluate the relationship between the expression of these cytokines and stem-like cell properties, we used the MDA-MB-231 cell line, a basal-type breast cancer cell line, and confirmed that these cells constitutively express IL6 and IL8 (Additional file 8: Figure S7A). These cells exhibit a spindle-like appearance and express high levels of CD44 in the absence of CD24 (Additional file 8: Figure S7B and C). As expected for a mesenchymal-type cell line MDA-MB-231 also expresses high levels of vimentin, ZEB1 and SNAIL-1, and low levels of E-cadherin, as evaluated by qRT-PCR (Additional file 8: Figure S7D). MDA-MB-231 cells showed less proliferation potential than MCF-7 cells (Additional file 8: Figure S7E), although Ki67 expression was slightly higher in the MDA-MD-231 cell line when evaluated during the exponential growth phase (3^rd^ day) (Additional file 8: Figure S7F, left panel), and not very different at day 5^th^ when cell growth slowed down (Additional file 8: Figure S7F, right panel). MDA-MB-231 cells spontaneously migrated more than the MCF-7 cells (Additional file 9: Figure S8A) and also showed higher directed migration towards FBS or IL8 (Additional file 9: Figure S8B). Accordingly, MDA-MB-231 cells presented higher invasion to matrigel-coated filters (Additional file 9: Figure S8C) and adherence to fibronectin (Additional file 9: Figure S8D) when compared to MCF-7 cells. Thus, MDA-MB-231 cells have an EMT phenotype and resemble MCF-7 cells that have been treated with cytokines or SCM. Interestingly, treatments with SCM did not impact morphology nor migration capacity of MDA-MB-231 cells, leaving open the question about the role of cytokines in the maintenance of these properties (Additional file 10: Figure S9A and B). We therefore sought to confirm the relevance of IL6 and IL8 in MDA-MB-231 properties, by either incubating these cells with neutralizing antibodies (Additional file 10: Figure S9) or by knocking down their expression (Additional file 11: Figure S10A). Inhibition of IL6 and IL8 by antibodies induced a morphological change in MDA-MB-231 cells (Additional file 10: Figure S9A) and a decrease in the migration capacity (Additional file 10: Figure S9B). The knockdown of IL6 or IL8 was associated with increased expression of the epithelial markers E-cadherin and TJP-1 (Additional file 11: Figure S10D). Also, the knockdown of IL6 and IL8 led to a decrease in the collagen adhesion capacity of MDA-MB-231 cells (Additional file 11: Figure S10B and S10C). These results support the idea that IL6 and IL8 play an important role and suggest that senescence/inflammation could be more relevant for the biology of cell lines that do not express these pro-inflammatory cytokines.

**Fig. 9:**
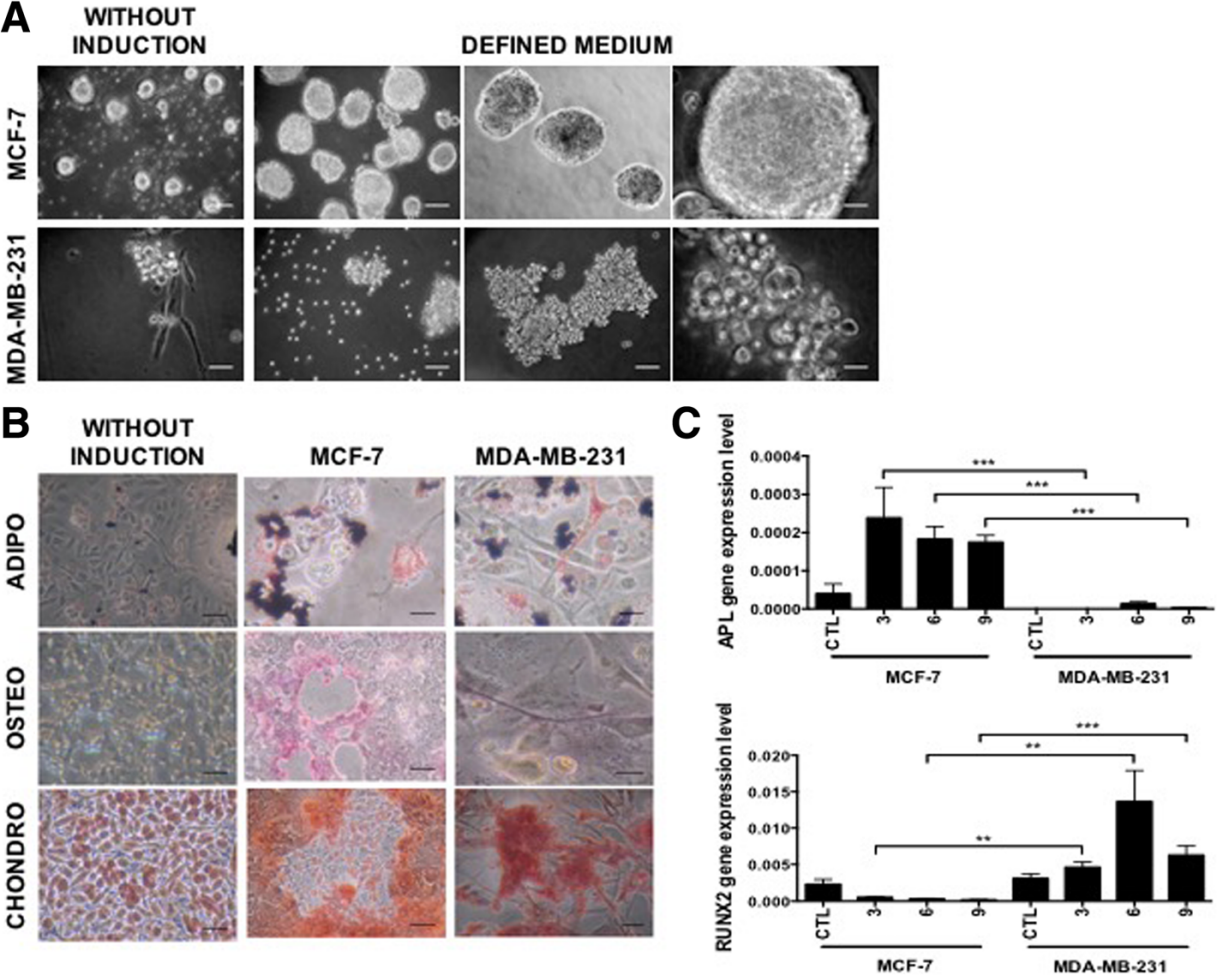
MDA-MB-231 cells exhibited incomplete/aberrant stem-like cell characteristics. **a** Sphere formation assay in the presence of defined medium (EGF and FGF) for the MCF-7 and MDA-MB-231 cell lines. Representative micrographs (10, 20 and 40×) show sphere formation after 9 days of induction. Scale bar, 100 μm (**b**). Differentiation assay of MCF-7 and MDA-MB-231 cells. Differentiation was evaluated after 10 days of induction. Osteoblasts (ALP activity), adipocytes (oil red-O) and chondrocytes (Safranin O) differentiation was determined with specific staining. Representative images are shown. Scale bar, 10 μm. **c** RUNX2 and APL gene expression determined at day 3, 6 and 9 of differentiation by qRT–PCR. The values were normalized to GADPH and relative to control cells. Error bars represent SEM (*p < 0.01; (**p < 0.001) (*n* = 2)

Next, we explored the concurrence of stem-like cell properties with the EMT process in the MDA-MB-231 cell line, as we have observed in cytokine-treated MCF-7, and as it has been reported in other cancer models [67–72]. Unexpectedly, cytokine pre-treated MDA-MB-231 cells do not form mammospheres, but instead cell aggregates, that were very irregular, translucent and of different size (Fig. 9a). This was in clear contrast to the high mammosphere-forming capacity of cytokine pre-treated MCF-7 cells, described above (Figs. 5a, c and 9a). In addition, although differentiation to chondro- and adipocytes was normal (Fig. 9b, upper and lower panels), MDA-MB-231 cells were surprisingly unable to differentiate into osteocytes (Fig. 9b, middle panel and Additional file 12: Figure S11A). MDA-MB-231 cells did not express APL upon osteocyte differentiation induction, in contrast to MCF-7 cells, (Fig. 9c). Intriguingly, MDA-MB-231 cells express high levels of RUNX2 (Fig. 9c), which can be an inhibitor of osteogenic differentiation. In relation to the expression of reprogramming TFs, there were no differences between MCF-7 and MDA-MB-231 (Additional file 12: Figure S11B).

In all, treatments of luminal A MCF-7 cell line with SCM, IL6 and IL8 were able to reveal an EMT phenotype, which is proper to basal/mesenchymal cell lines like MDA-MB-231, as well as CSC-like properties. However, it appears that both cell lines differ in important ways regarding their stem-like cell capabilities suggesting that EMT and CSC properties are not always acquired together.

## Discussion

Previous work has shown that fibroblast-derived SASP induced a classic EMT in two human non-aggressive luminal breast cancer cell lines T47D and ZR75.1 and that this effect was principally exerted through IL6 and IL8 secretion [18]. These inflammatory cytokines are consistently present in SASP [16, 18] suggesting the existence of a common inflammatory effect associated with senescence in pre-malignant and malignant cells. We have previously shown that SCM from senescent HCA2 fibroblasts is partially responsible for unveiling epithelial plasticity and CSC-like properties in premalignant cells [15]. We have shown here that fibroblast SCM is highly enriched in IL6 and IL8 and that these cytokines were capable of inducing an EMT-like program in MCF-7, another luminal breast cancer cell line. Nevertheless, this EMT program was only partial with CD44 expression increasing in a fraction of the cell population and with other EMT-associated markers showing variable responses with the different treatments. This suggests the existence of differentiation plasticity in MCF-7 cells, as previously observed in the HEK model [15]. In spite of differences in gene expression, MCF-7 cells consistently adopted a mesenchymal cell morphology associated with higher migration potential towards FBS or IL8, higher EMT-associated TFs expression and increased adherence to fibronectin. We argue, by analogy to the HEK model, that SCM-treated MCF-7 cells adopt a metastable state that help reveal particular/specific cell competencies.

Indeed, the cytokine-induced EMT program was accompanied by a concomitant appearance of cells with stem-like cell properties as reported by other authors using different inducers [50, 51]. We have shown here, that both inflammatory cytokines IL6 and IL8 have the capability to induce typical stem-like cell properties (self-renewal and differentiation capabilities) associated with malignity in otherwise low aggressive breast cancer cells. The importance of this observation is underlined by the widely accepted view that a tumorigenic phenotype may be initiated and preserved in a subpopulation of cells that shows stem-like cell properties ([73–75]. Remarkably, luminal A MCF-7 breast cancer cells, in spite of its lower tumorigenic potential, are intrinsically capable of displaying stem-like cell properties (low basal sphere-forming capacity and some differentiation potential) but these properties are substantially strengthened by a senescence-associated inflammatory microenvironment. It is important to note that the development of stem-like cell properties was highly consistent in response to the cytokine treatments, as opposed to the induction of a full-blown EMT program.

The role of IL6 or IL8 has been independently studied in breast cancer lines due to their different roles in tumour biology, the different cellular origins from which they can be obtained and their cancer-related inflammatory roles [76–79]. For instance IL6 effect has been studied in the dynamic conversion between CSC and non-CSC [10, 26, 27] or its effect on the maintenance and enrichment of the CSC population using the in vitro mammosphere-forming assay [29]. On the other hand and due to its chemokine role, IL8 has been studied in the context of migration and invasion in breast cancer [80] and more recently, it has also been associated with CSC and metastasis [34]. As mentioned above, we observed differences between the treatments with IL6 or IL8, in special in the induction of the EMT program. Interestingly, IL8 seems to better simulate the observed effect with the SCM and consistently induced characteristics associated with CSC. Unexpectedly, this effect was not enhanced by the addition of IL6, in spite of the fact that IL6 has similar effects to IL8; rather, it seems that IL6 can paradoxically inhibit, at least in some instances, the IL8 effect (for example, EMT-TFs (Fig. 4a), the reprogramming TF Nanog (Fig. 6b), SA-β-GAL induction (Fig. 8c) and p21 expression (Fig. 8e). The significance of this is unknown to us but it would be interesting to ascertain if there is any connection between these different observed effects in the context of inflammatory cytokines. Whether it is the same cell subpopulation that responds to IL6 or to IL8 remains to be determined.

We have shown that SCM can induce the secretion of IL6 and IL8 by MCF-7 cells, suggesting that a reinforcing inflammatory loop can be established. This has also been described for IL8 in other models [18]. Interestingly, exposure to IL6 or IL8 induced SA-β-GAL activity in MCF-7 cells creating a second loop that could contribute to reinforce and spread the effect of the senescence/inflammatory microenvironment. This paracrine-induced senescence has also been shown recently for TGF-β, VEGF and CCL2 suggesting that this is a general mechanism for senescence propagation involving not only tumour cells but also surrounding stromal cells [81]. Of note, the simultaneous addition of IL6 and IL8 or TGF-β at higher concentration to MCF-7 cells did not induce senescence after 5 days of exposure. On the contrary, addition of a hundred times lower concentration of both IL6 and IL8 cytokines for a prolonged period of time (10 days) induced the appearance of SA-β-GAL positive MCF-7 cells. Clearly, a self- and cross- reinforced senescence/inflammatory microenvironment where cells are chronically exposed to such soluble factors has potential physiological relevance.

For instance, the presence of a low proportion of senescent-cell progenitors in the MCF-7 cell line [44] may create the basal condition that initiates the production of reinforcing stimuli leading to the formation of foci of senescent cells surrounded by tumour and inflammatory cells [82]. Accordingly, it has been shown that cytokines might reinforce a senescence arrest only when cells are moderately damaged or near senescent [83]. In this senescent/inflammatory self-reinforcing condition, luminal breast cancer cells showing low aggressiveness could turn into highly tumorigenic cells.

The situation seems to be different for the MDA-MB-231 basal/mesenchymal breast cancer cell line. First, this cell line has been classified as immortalized-cell progenitor subtype, without a senescent component. The absence of senescent-cell progenitors would compromise the establishment of the senescence reinforcing loops. Second, MDA-MB-231 cell line, which secrets high amounts of IL6 and IL8, is already pre-set to a basal/mesenchymal program, and expresses constitutively malignant features associated with higher tumorigenicity. These features include high migration and invasion capacities and, presumably, the presence of stem-like cell properties, such as self-renewal and differentiation potential. Our work showed that IL6 and IL8 production are indeed important in the maintenance of EMT-like features. However, to our surprise, we found that MDA-MB-231 had no sphere-forming capacity and this inability was not modified by the addition of either cytokines or neutralizing antibodies to these cytokines (not shown). The reasons for this observation are unknown and warrant a better evaluation. On the other hand, MDA-MB-231 cells showed reduced osteogenic differentiation potential, when compared to MCF-7 cells. Further evaluation showed that the aberrant capacity to differentiate of the MDA-MB-231 cells was associated to a lower expression of APL compared to MCF-7 cells. On the other hand, MCF-7 showed a similar APL expression kinetics to mesenchymal stem cells [84]. Additionally, MDA-MB-231 showed an increase of RUNX2, a known transcription factor required for osteoblast lineage definition in pre-osteoblast, but an inhibitor of the osteoblastic maturation and mature bone formation [85, 86]. Although the significance of this abnormal osteogenic differentiation remains unclear, it is believed that aberrant differentiation contributes to breast cancer heterogeneity [87]. One could envisage that part of the aggressiveness of basal/mesenchymal breast cancer cells is more related to this abnormal differentiation-induced cell heterogeneity than to the stem-like cell capabilities they may have. Consequently with this, MCF-7 breast cancer cells have basal stem-like cell capabilities (self-renewal and differentiation potential), though have a less aggressive tumorigenic potential.

We have shown here that a senescence-induced inflammatory microenvironment, enriched in the cytokines IL6 and IL8, is responsible for a prevailing inflammatory-senescence response that persists for longer periods because of their self and cross reinforcing characteristics. This continuous stimulation potentiates epithelial plasticity with acquisition of stem-like cells properties that could transform non-aggressive breast cancer cells (MCF-7) in cells having a more tumorigenic potential. It would be interesting to decipher the real contribution of cell heterogeneity and stem-like cell capabilities to cancer aggressiveness, and the role that the tumour microenvironment may have in these cell properties.

## Conclusions

Our findings show that the IL-6 and IL-8 present in the SCM are responsible for the induction of epithelial plasticity and stemness in the malignant MCF-7 cells. Endogenous IL-6 and IL-8 production is also responsible for the EMT-like behaviour of the more aggressive MDA-MB-231 cell line. However, these cells showed abnormal differentiation and incomplete stemness, suggesting that in this case aggressiveness is more related to EMT properties than to stem-like cell properties. All in all, our results underscore that stemness, although necessary for tumorigenicity, is varied, differentially influenced by the senescent/inflammatory microenvironment and contributes distinctly to the aggressiveness of tumour cells.

## Additional files

Additional file 1: Table S1. Primer sequence. (DOCX 29 kb)

Additional file 2: Figure S1. The SCM is rich in the pro-inflammatory cytokines IL6 and IL8. (A) Representative images (10× and 40×) of cells stained for SA-β-GAL. Scale bar 10 μm. (B) Pro-inflammatory cytokines, IL6 and IL8 were measured by using a Becton Dickinson Cytometric Bead Array (CBA) flow cytometric assay, using free serum conditioned medium from young and senescent fibroblast. Bar graph shows the amount of cytokine in pg/ml. (C) Gene expression levels of EMT markers and EMT TFs and (D) reprogramming TFs, determined by qRT-PCR. The values were normalized to GADPH and relative to control cells (dotted lines). Error bars represent SEM. (*p < 0.05; **p < 0.01; ***p < 0.001) (*n* = 2). (DOCX 632 kb)

Additional file 3: Figure S2. The treatment with IL6 and IL8 induces an increase in CD44 expression. (A) CD44 gene expression in MCF-7 cells stimulated with cytokines as indicated. The values were normalized to GADPH and relative to control cells (dotted lines). Error bars represent SEM. (**p < 0.01) (*n* = 2). (B) BrdU incorporation of MCF-7 cells treated or not with cytokines during the migration assay (12 h time point). Cells were incubated with 5-bromo-2′deoxyuridine (30 μM) for 25 min and stained with DAPI and analysed by FACS. The percentage of cells in the different cell-cycle phases is shown. (DOCX 857 kb)

Additional file 4: Figure S3. The treatment with IL6 and IL8 induces slight changes in the proliferation of MCF-7 cells and in the expression of EMT markers. (A) Growth kinetics of MCF-7 cells upon treatment with cytokines. Equal numbers of cells were seeded in triplicate and treated with either control normal or cytokines-supplemented medium. Cells were counted at the indicated time points. Error bars represent SEM. ***p < 0.001 indicate statistically significant differences between control cells and cytokine treatments. (*n* = 2). (B) Gene expression profile of EMT-associated transcription factors in MCF-7 cells stimulated with cytokines as indicated and determined by qRT–PCR. The values were normalized to GADPH and relative to control cells (dotted lines). Error bars represent SEM. (**p < 0.01) (*n* = 2). ECADH = E-cadherin, KR18 = cytokeratin 18, VIM = vimentin y TJP1 = tight junction protein 1. (DOCX 236 kb)

Additional file 5: Figure S4. MCF-7 cells treated with IL6 and IL8 exhibit self-renewal properties. (A) Sphere formation assay in the presence of defined medium (EGF and FGF) in MCF-7 cells that were previously treated or not (Control, CTL) with cytokines. The total number of spheres per well larger than 100 μm was counted after the treatment with IL-6 and IL-8 during 3 or 4 days, as indicated. Error bars indicate SEM. (**p < 0.01) (*n* = 3). (B) Representative micrographs (10×) showing sphere fusion after 4 days of induction, scale bar, 100 μm. (C) The total number of primary, secondary and tertiary spheres per well larger than 100 μm was counted at day 5. Error bars indicate SEM. (***p < 0.001; **p < 0.01; *p < 0.05) (*n* = 2). (DOCX 587 kb)

Additional file 6: Figure S5. The pro-inflammatory cytokines IL6 and IL8 are important players in the EMT process. (A) Neutralization of IL6 and IL8 with 1 μg/ml of monoclonal antibodies against IL6 (Ref: MAB206, R&D systems) or IL8 (Ref: AF-208-NA, R&D systems). The levels of IL6 and IL8 were measured by using a Becton Dickinson Cytometric Bead Array (CBA) flow cytometric assay, using free serum conditioned medium from MCF-7 that were treated with SCM or with SCM plus neutralizing antibodies specific for each cytokine. Bar graph shows the amount of cytokine in pg/ml before and after treatment. (B) Gene expression levels of EMT TFs (B) and reprogramming TFs (C). The values were normalized to GADPH and relative to control cells (dotted lines). Error bars represent SEM. (DOCX 623 kb)

Additional file 7: Figure S6. The SCM increases the expression of IL6 and IL8 in MCF-7 cells that was accompanied by an irreversible senescence state. (A) Representative images (40×) of MCF-7 cells treated with SCM during 5 days or (C) with cytokines at low concentrations (0,5 ng/ml) during 10 days and stained for SA-β-GAL. Scale bar, 10 μm. (B) IL6 and IL8 were measured by CBA flow cytometric assay using free serum conditioned medium from MCF-7 treated as indicated above. (D) BrdU incorporation detected in MCF-7 cells treated with SCM during 5, 7 or 10 days to induce senescence and after additional incubation with growth medium (RPMI-1640 and 10% SFB) for 48 h. The histogram shows the ratio between S and G1 phases of cell cycle (left). Error bars indicate SEM. Representative images (10×) of MCF-7 cells stained for SA-β-GAL (Right). (E) Transwell migration assay towards 50 ng/μl of IL-8 performed in MCF-7 cells treated or not (control) with SCM during 5 days. Representative micrographs (20×) taken from the membrane filter (bottom surface of filters) stained with Crystal violet. Cells present in the lower compartment were counted. Error bars indicate SEM. (**p < 0.01). (DOCX 237 kb)

Additional file 8: Figure S7. MDA-MB-231 cells are rich in pro-inflammatory cytokines and display an EMT-like phenotype. (A) Pro-inflammatory cytokines, IL6 and IL8 were measured by CBA flow cytometric assay, using free serum conditioned medium from MCF-7 and MDA-MB-231 cells. (B) Morphological evaluations by phase contrast microscopy (20 ×). Arrowhead indicates cells with fibroblastoid morphology. Scale bar, 10 μm. (C) Surface markers expression of CD44 and CD24 was determined by FACS as above or by qRT-PCR. (D) Gene expression of EMT-associated markers was evaluated by qRT–PCR. The histogram shows the expression of these markers relative to GADPH. Error bars indicate SEM. (***P < 0.001). E-cadherin and TJP1 (epithelial markers) and Vimentin, Zeb1, Twist1, Snail1 and Snail2/Slug (mesenchymal markers). (E) Growth kinetics of MCF-7 and MDA-MB-231 cell lines. Error bars represent SEM. (***p < 0.001) (*n* = 2). (F) Representative FACS histograms showing the Ki-67 analysis done at day 3 and 5 (Left and right histograms, respectively). MDA-MB-231 cells (gray line), MCF-7 cells (black line), blanc (filled histogram) and isotype control (dotted line). (DOCX 556 kb)

Additional file 9: Figure S8. MDA-MB-231 exhibits high migration capacity. (A) Wound healing assays were performed in MCF-7 and MDA-MB-231 cells. Representative micrographs (10×) were taken at 0, 16, and 24 h and showed an increased migration capacity of MDA-MB-231 cells (left). Black dotted line indicated the area of the wound. Quantification of wound healing assay from at least 10 images (right) by using the image J program. Error bars indicate SEM. (***p < 0.001) (*n* = 2). (B) Transwell migration assay performed in MCF-7 and MDA-MB-231 cells by using SFB (20%) or IL8 (50 ng/μl) as chemoattractants in the lower compartment. Error bars indicate SEM. (***p < 0.001). (C) Matrigel invasion assay; cells were allowed to invade during 48 h at 37 °C. Representative micrographs (20×) were taken from the upper panel (bottom surface of filters) and from the lower compartment (LC) and stained with violet Crystal. The histograms show the number of cells in the lower compartment. Error bars indicate SEM. (**p < 0.01) (*n* = 2). (D) Adhesion of MDA-MB-231 and MCF-7 cells to 96 multiwell plates coated with fibronectin. Representative micrographs (20×) were taken from the plates and stained with violet Crystal (left). The number of adherent cells was counted. Error bars indicate SEM. (*p < 0.05) (*n* = 3). (DOCX 646 kb)

Additional file 10: Figure S9. Il6 and IL8 are relevant in the acquisition of functional properties of the EMT process in MDA-MB-231. MDA-MB-231 cells were treated or not (Control) with SCM or with 1 μg/ml of neutralizing monoclonal antibodies or IgG isotype control in RPMI medium without serum. (A) The morphological evaluation was done at 72 h after treatment. Representative images (10 and 20 ×) are shown. Scale bar, 10 μm. (B) Wound healing assays were performed in MDA-MB-231 cells. Representative micrographs (10×) were taken at 0, 4, 8 and 12 h and showed a decrease in migration capacity of MDA-MB-231 cells (upper). Black dotted line indicated the area of the wound. Quantification of wound healing assay from at least 10 images (bottom) by using the image J program. Error bars indicate SEM. (***p < 0.001) (*n* = 2). (DOCX 268 kb)

Additional file 11: Figure S10. Il6 and IL8 are important players in the acquisition of characteristics associated to EMT in MDA-MB-231. The knockdown of the pro-inflammatory cytokines was carried out by using esiRNA for IL8 and shRNA for IL6. The esiRNA-FLUC and shRNA Puro vector were used as control, respectively. (A) The decrease in the expression of these cytokines and (D) EMT markers was confirmed by qRT–PCR. The values were normalized to GADPH and relative to control cells (dotted lines). Error bars represent SEM. (*p < 0.05; **p < 0.01***p < 0.001). Adhesion of normal or IL6 and IL8 knockdown MDA-MB-231to 96 multiwell plates coated collagen. Representative micrographs (10×) were taken from the plates and stained with Crystal violet (B). The number of adherent cells was counted. Error bars indicate SEM. (***p < 0.001). (DOCX 578 kb)

Additional file 12: Figure S11. MDA-MB-231 cannot differentiate to osteogenic lineage and are similar to MCF-7 in the expression of reprograming factors. (A) Differentiation was promoted towards the osteogenic lineage by using specific induction medium. Differentiation was evaluated after 10 days with specific staining (APL activity). Representative images are shown (10, 20 and 40 ×). Scale bar, 10 μm. Cells without induction medium were used as a control. (B) Gene expression levels of reprogramming-associated transcription factors were evaluated by qRT–PCR. The values were normalized to GADPH. Error bars represent SEM. (DOCX 1247 kb)

## Abbreviations

APL: Alkaline phosphatase
CBA: BD™ cytometric bead array
CSC: Cancer stem cells
EMT: Epithelial-mesenchymal transition
ER+/PR+: Estrogen and progesterone receptor positive
FACS: Fluorescence-activated cell sorting
FBS: Fetal bovine serum
FC: κ isotype Ctrl
Her2-: Her-2/neu negative
SA-βGal: Senescence-associated beta-galactosidase
SASP: Senescence-associated secretory phenotype
SCM: senescent-conditioned medium
TFs: Transcription factors
