# Supplementary material for: Senescence-associated IL-6 and IL-8 cytokines induce a self- and cross-reinforced senescence/inflammatory milieu strengthening tumorigenic capabilities in the MCF-7 breast cancer cell line
Source: Cell Commun Signal. 2017 May 4;15:17. doi: 10.1186/s12964-017-0172-3 (PMC5418812; doi:10.1186/s12964-017-0172-3)
Supplement: Supplementary file 1 — Primer sequence. (DOCX 29 kb) [file 12964_2017_172_MOESM1_ESM.docx]

**Additional file 1: Table S1. Primer sequence**

| **Name** | **F-Sequence** | **R- Sequence** |
| --- | --- | --- |
| **Epithelial markers** | | |
| **E-CADHERINA** | TGGACAGGGAGGATTTTGAG | ACCCACCTCTAAGGCCATCT |
| **TJP1** | ACAAAGGAGAGGTGTTCCGTGTT | CGTTCTACCTCCTTATGATTTTTACCA |
| **KR18** | AGCTCAACGGGATCCTGCTGCACCTTG | CACTATCCGGCGGGTGGTGGTCTTTTG |
|  |  |  |
| **Mesenchymal markers** | | |
| **CD44** | TGCCGCTTTGCAGGTGTAT | GGCCTCCGTCCGAGAGA |
| **VIMENTINA** | CGAGGACGAGGAGAGCAGGATTTCTC | GGTATCAACCAGAGGGAGTGA |
| **ZEB1** | AAGAATTCACAGTGGAGAGAAGCCA | GGTTTCTTGCAGTTTGGGCATT |
| **TWIST 1** | GGAGTCCGCAGTCTTACGAG | TCTGGAGGACCTGGTAGAGG |
| **TWIST 2** | CTTATGTTTGGGGGGAGGTT | TAGCCAAGCAATCACGGAGA |
| **SNAIL 1** | \| ACCACTATGCCGCGCTCTT \|  \| \| --- \| --- \| | GGTCGTAGGGCTGCTGGAA |
| **SNAIL 2** | TGTTGCAGTGAGGGCAAGAA | GACCCTGGTTGCTTCAAGGA |
| **Reprogramming markers** | | |
| **OCT4** | AGTTTGTGCCAGGGTTTTTG | CTTCACCTTCCCTCCAACC |
| **NANOG** | CCTGTGATTTGTGGGCCT | GACAGTCTCCGTGTGAGGCAT |
| **SOX2** | GTATCAGGAGTTGTCAAGGCAGAG | TCCTAGTCTTAAAGAGGCAGCAAAC |
| **KLF4** | TATGACCCACACTGCCAGAA | TGGGAACTTGACCATGATTG |
| **Cytokines** | | |
| **IL6** | GGAGACTTGCCTGGTGAAAA | GTCAGGGGTGGTTATTGCAT |
| **IL8** | TTGGCAGCCTTCCTGATTTC | AACTTCTCCACAACCCTCTGCA |
| **Housekeeping** | | |
| **GADPH** | GACCCCTTCATTGACCTCAAC | CTTCTCCATGGTGGTGAAGA |
