# Supplementary figures and images for: Senescence-associated IL-6 and IL-8 cytokines induce a self- and cross-reinforced senescence/inflammatory milieu strengthening tumorigenic capabilities in the MCF-7 breast cancer cell line
Source: Cell Commun Signal. 2017 May 4;15:17. doi: 10.1186/s12964-017-0172-3 (PMC5418812; doi:10.1186/s12964-017-0172-3)

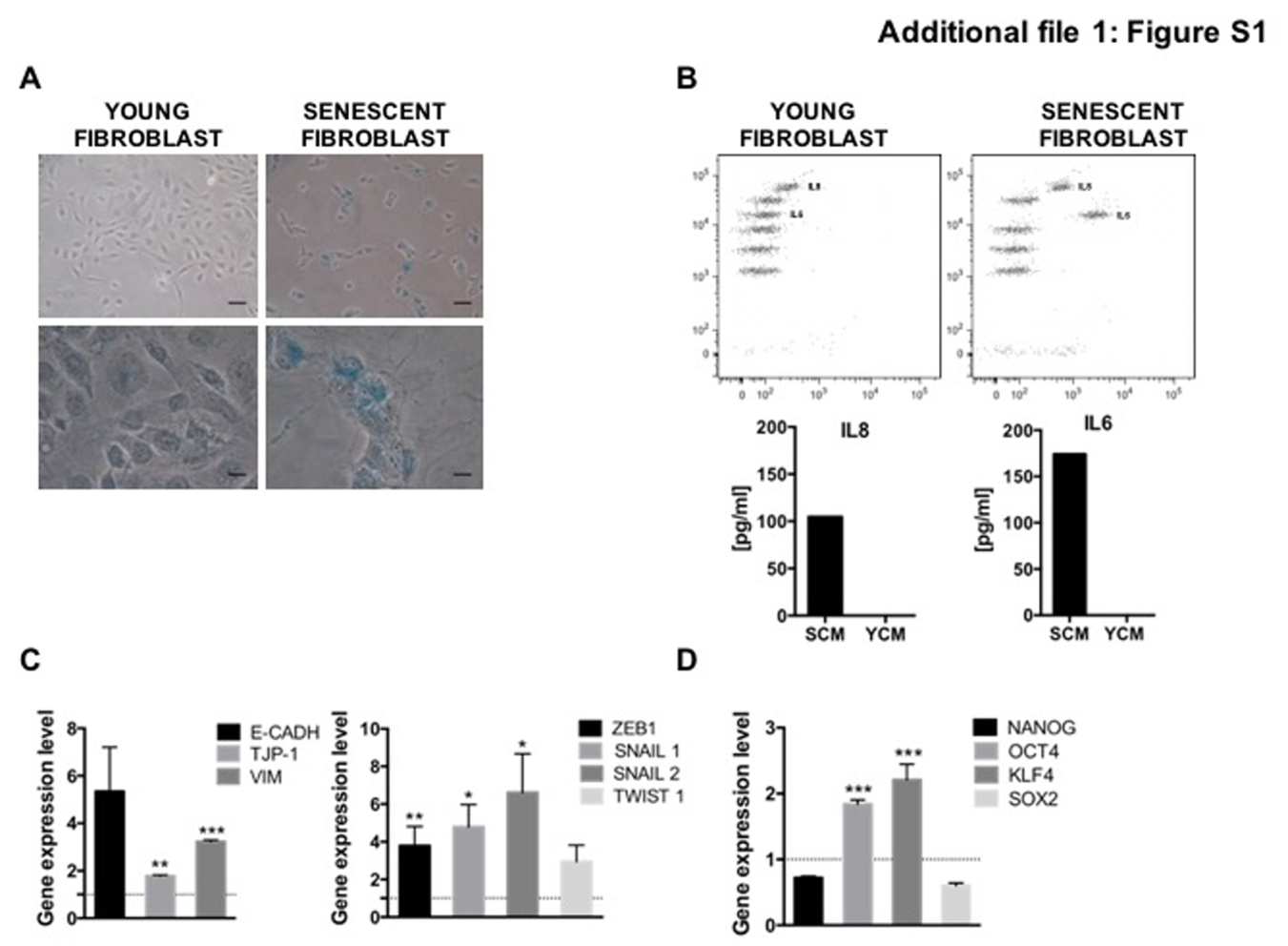

Supplement: Supplementary file 2 — The SCM is rich in the pro-inflammatory cytokines IL6 and IL8. (A) Representative images (10× and 40×) of cells stained for SA-β-GAL. Scale bar 10 μm. (B) Pro-inflammatory cytokines, IL6 and IL8 were measured by using a Becton Dickinson Cytometric Bead Array (CBA) flow cytometric assay, using free serum conditioned medium from young and senescent fibroblast. Bar graph shows the amount of cytokine in pg/ml. (C) Gene expression levels of EMT markers and EMT TFs and (D) reprogramming TFs, determined by qRT-PCR. The values were normalized to GADPH and relative to control cells (dotted lines). Error bars represent SEM. (*p < 0.05; **p < 0.01; ***p < 0.001) (n = 2). (DOCX 632 kb) [file 12964_2017_172_MOESM2_ESM.docx]

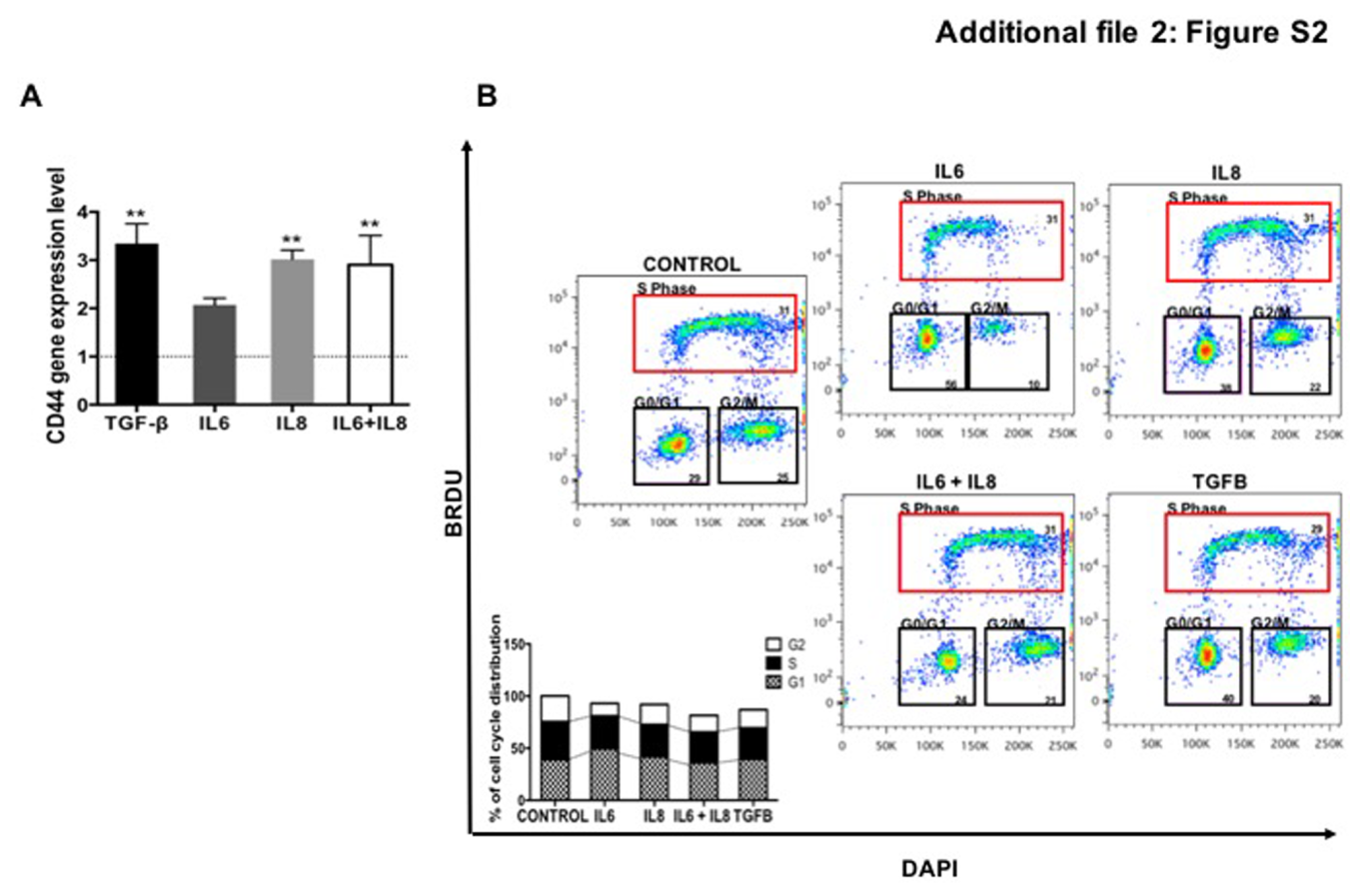

Supplement: Supplementary file 3 — The treatment with IL6 and IL8 induces an increase in CD44 expression. (A) CD44 gene expression in MCF-7 cells stimulated with cytokines as indicated. The values were normalized to GADPH and relative to control cells (dotted lines). Error bars represent SEM. (**p < 0.01) (n = 2). (B) BrdU incorporation of MCF-7 cells treated or not with cytokines during the migration assay (12 h time point). Cells were incubated with 5-bromo-2′deoxyuridine (30 μM) for 25 min and stained with DAPI and analysed by FACS. The percentage of cells in the different cell-cycle phases is shown. (DOCX 857 kb) [file 12964_2017_172_MOESM3_ESM.docx]

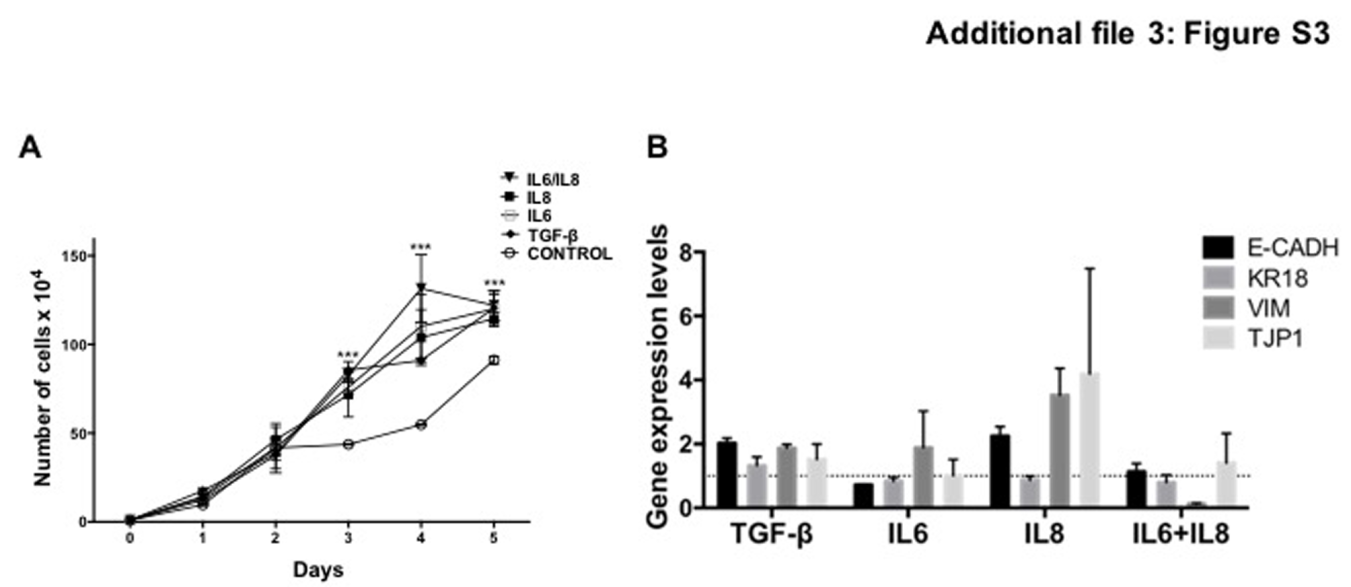

Supplement: Supplementary file 4 — The treatment with IL6 and IL8 induces slight changes in the proliferation of MCF-7 cells and in the expression of EMT markers. (A) Growth kinetics of MCF-7 cells upon treatment with cytokines. Equal numbers of cells were seeded in triplicate and treated with either control normal or cytokines-supplemented medium. Cells were counted at the indicated time points. Error bars represent SEM. ***p < 0.001 indicate statistically significant differences between control cells and cytokine treatments. (n = 2). (B) Gene expression profile of EMT-associated transcription factors in MCF-7 cells stimulated with cytokines as indicated and determined by qRT–PCR. The values were normalized to GADPH and relative to control cells (dotted lines). Error bars represent SEM. (**p < 0.01) (n = 2). ECADH = E-cadherin, KR18 = cytokeratin 18, VIM = vimentin y TJP1 = tight junction protein 1. (DOCX 236 kb) [file 12964_2017_172_MOESM4_ESM.docx]

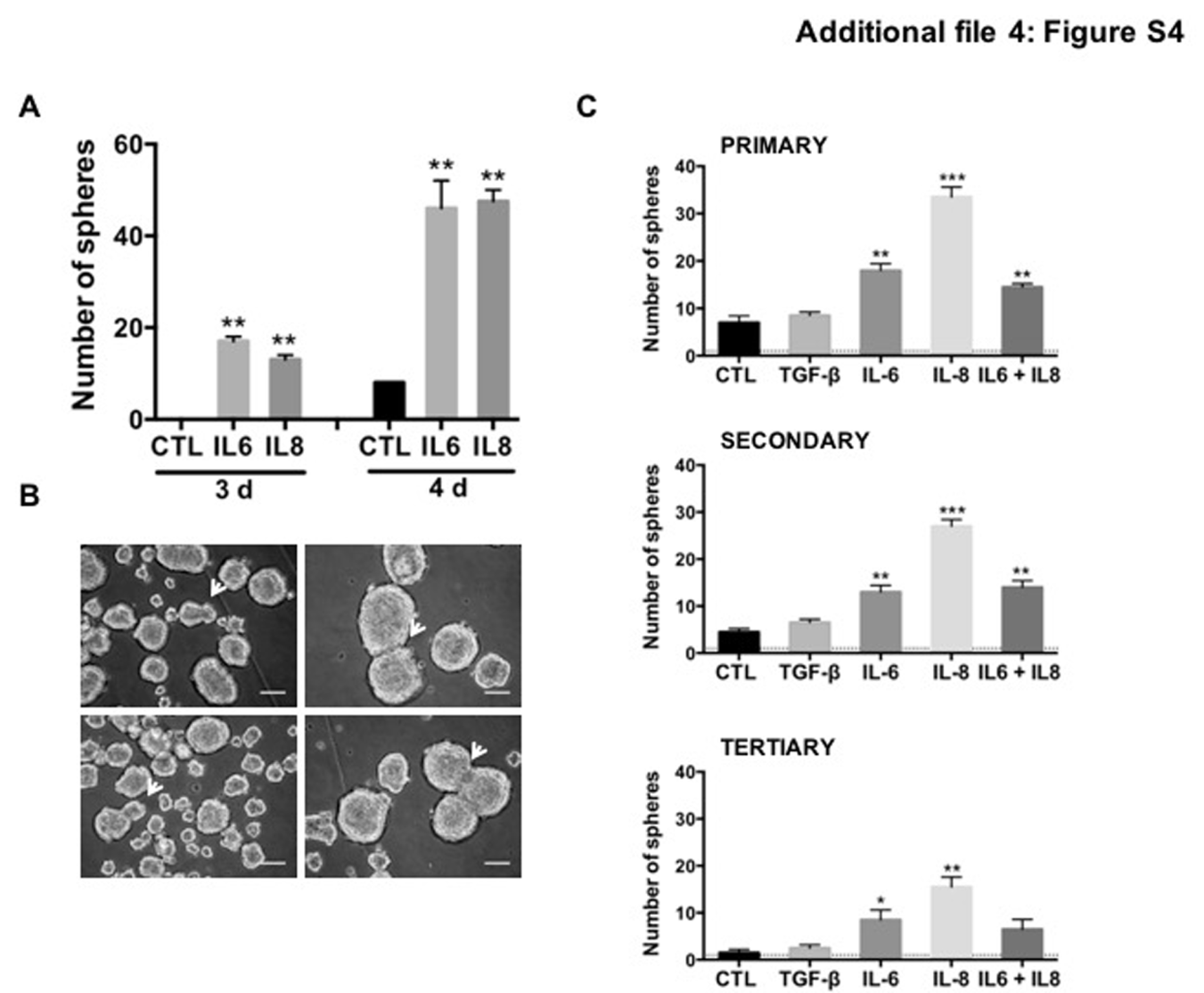

Supplement: Supplementary file 5 — MCF-7 cells treated with IL6 and IL8 exhibit self-renewal properties. (A) Sphere formation assay in the presence of defined medium (EGF and FGF) in MCF-7 cells that were previously treated or not (Control, CTL) with cytokines. The total number of spheres per well larger than 100 μm was counted after the treatment with IL-6 and IL-8 during 3 or 4 days, as indicated. Error bars indicate SEM. (**p < 0.01) (n = 3). (B) Representative micrographs (10×) showing sphere fusion after 4 days of induction, scale bar, 100 μm. (C) The total number of primary, secondary and tertiary spheres per well larger than 100 μm was counted at day 5. Error bars indicate SEM. (***p < 0.001; **p < 0.01; *p < 0.05) (n = 2). (DOCX 587 kb) [file 12964_2017_172_MOESM5_ESM.docx]

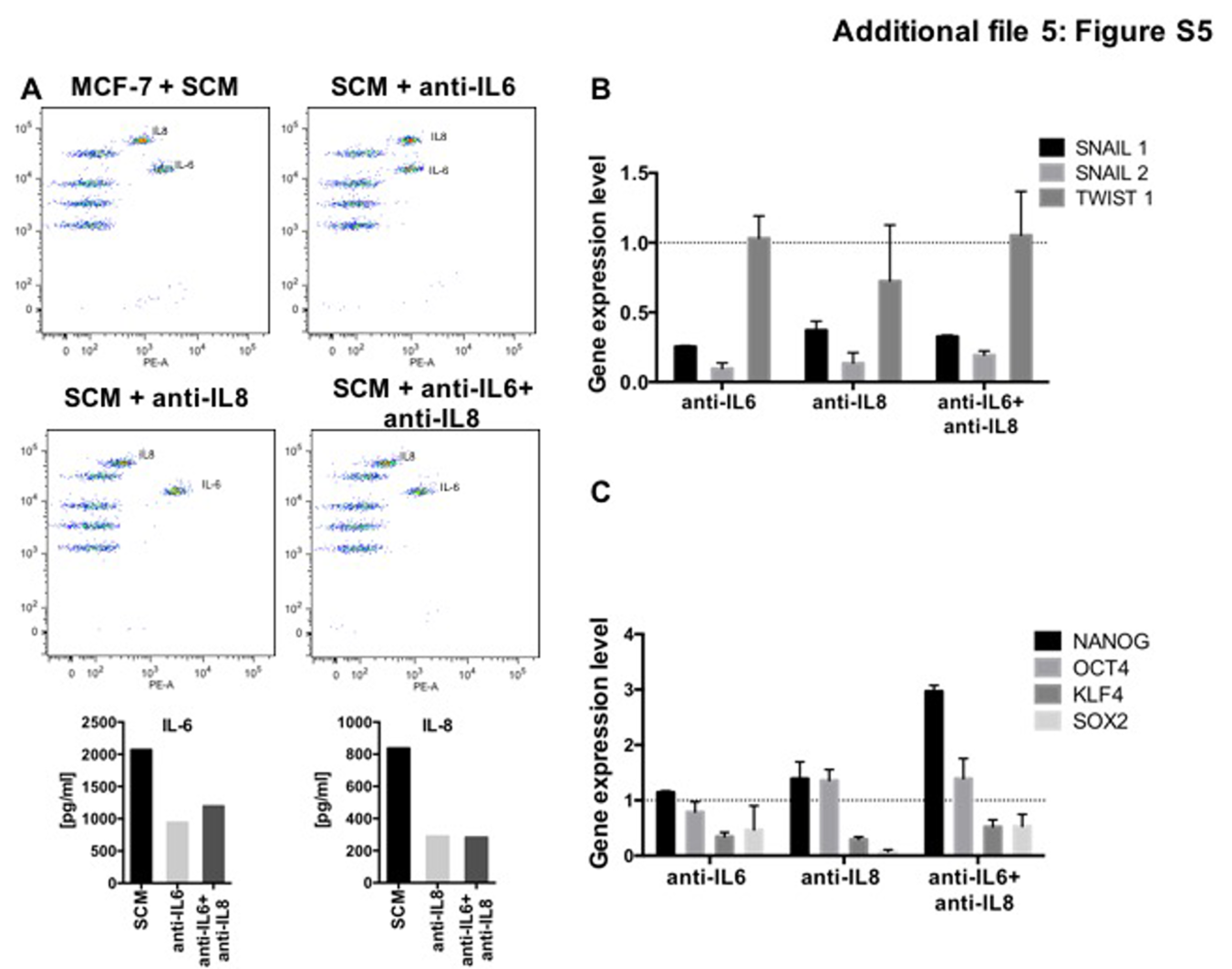

Supplement: Supplementary file 6 — The pro-inflammatory cytokines IL6 and IL8 are important players in the EMT process. (A) Neutralization of IL6 and IL8 with 1 μg/ml of monoclonal antibodies against IL6 (Ref: MAB206, R&D systems) or IL8 (Ref: AF-208-NA, R&D systems). The levels of IL6 and IL8 were measured by using a Becton Dickinson Cytometric Bead Array (CBA) flow cytometric assay, using free serum conditioned medium from MCF-7 that were treated with SCM or with SCM plus neutralizing antibodies specific for each cytokine. Bar graph shows the amount of cytokine in pg/ml before and after treatment. (B) Gene expression levels of EMT TFs (B) and reprogramming TFs (C). The values were normalized to GADPH and relative to control cells (dotted lines). Error bars represent SEM. (DOCX 623 kb) [file 12964_2017_172_MOESM6_ESM.docx]

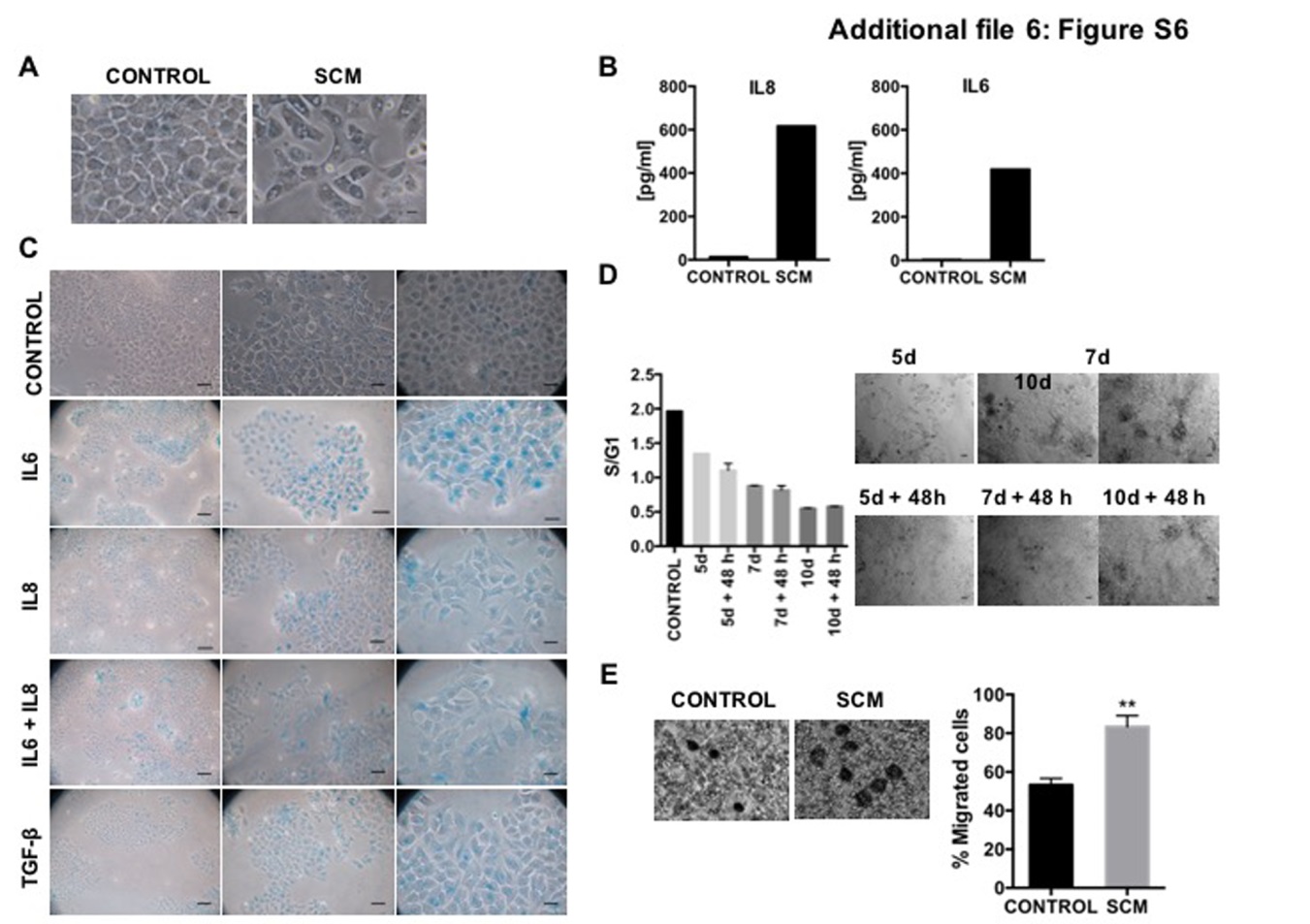

Supplement: Supplementary file 7 — The SCM increases the expression of IL6 and IL8 in MCF-7 cells that was accompanied by an irreversible senescence state. (A) Representative images (40×) of MCF-7 cells treated with SCM during 5 days or (C) with cytokines at low concentrations (0,5 ng/ml) during 10 days and stained for SA-β-GAL. Scale bar, 10 μm. (B) IL6 and IL8 were measured by CBA flow cytometric assay using free serum conditioned medium from MCF-7 treated as indicated above. (D) BrdU incorporation detected in MCF-7 cells treated with SCM during 5, 7 or 10 days to induce senescence and after additional incubation with growth medium (RPMI-1640 and 10% SFB) for 48 h. The histogram shows the ratio between S and G1 phases of cell cycle (left). Error bars indicate SEM. Representative images (10×) of MCF-7 cells stained for SA-β-GAL (Right). (E) Transwell migration assay towards 50 ng/μl of IL-8 performed in MCF-7 cells treated or not (control) with SCM during 5 days. Representative micrographs (20×) taken from the membrane filter (bottom surface of filters) stained with Crystal violet. Cells present in the lower compartment were counted. Error bars indicate SEM. (**p < 0.01). (DOCX 237 kb) [file 12964_2017_172_MOESM7_ESM.docx]

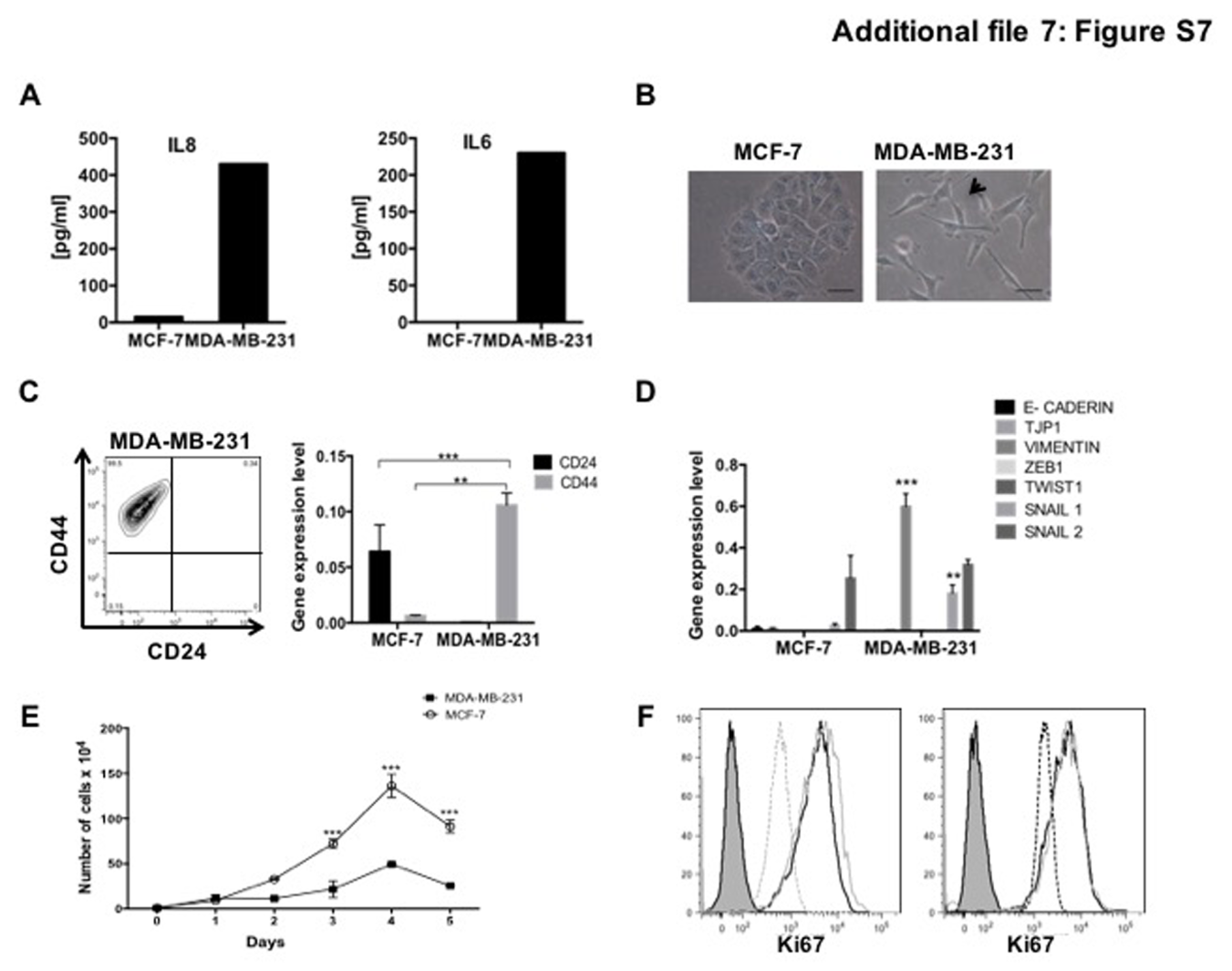

Supplement: Supplementary file 8 — MDA-MB-231 cells are rich in pro-inflammatory cytokines and display an EMT-like phenotype. (A) Pro-inflammatory cytokines, IL6 and IL8 were measured by CBA flow cytometric assay, using free serum conditioned medium from MCF-7 and MDA-MB-231 cells. (B) Morphological evaluations by phase contrast microscopy (20 ×). Arrowhead indicates cells with fibroblastoid morphology. Scale bar, 10 μm. (C) Surface markers expression of CD44 and CD24 was determined by FACS as above or by qRT-PCR. (D) Gene expression of EMT-associated markers was evaluated by qRT–PCR. The histogram shows the expression of these markers relative to GADPH. Error bars indicate SEM. (***P < 0.001). E-cadherin and TJP1 (epithelial markers) and Vimentin, Zeb1, Twist1, Snail1 and Snail2/Slug (mesenchymal markers). (E) Growth kinetics of MCF-7 and MDA-MB-231 cell lines. Error bars represent SEM. (***p < 0.001) (n = 2). (F) Representative FACS histograms showing the Ki-67 analysis done at day 3 and 5 (Left and right histograms, respectively). MDA-MB-231 cells (gray line), MCF-7 cells (black line), blanc (filled histogram) and isotype control (dotted line). (DOCX 556 kb) [file 12964_2017_172_MOESM8_ESM.docx]

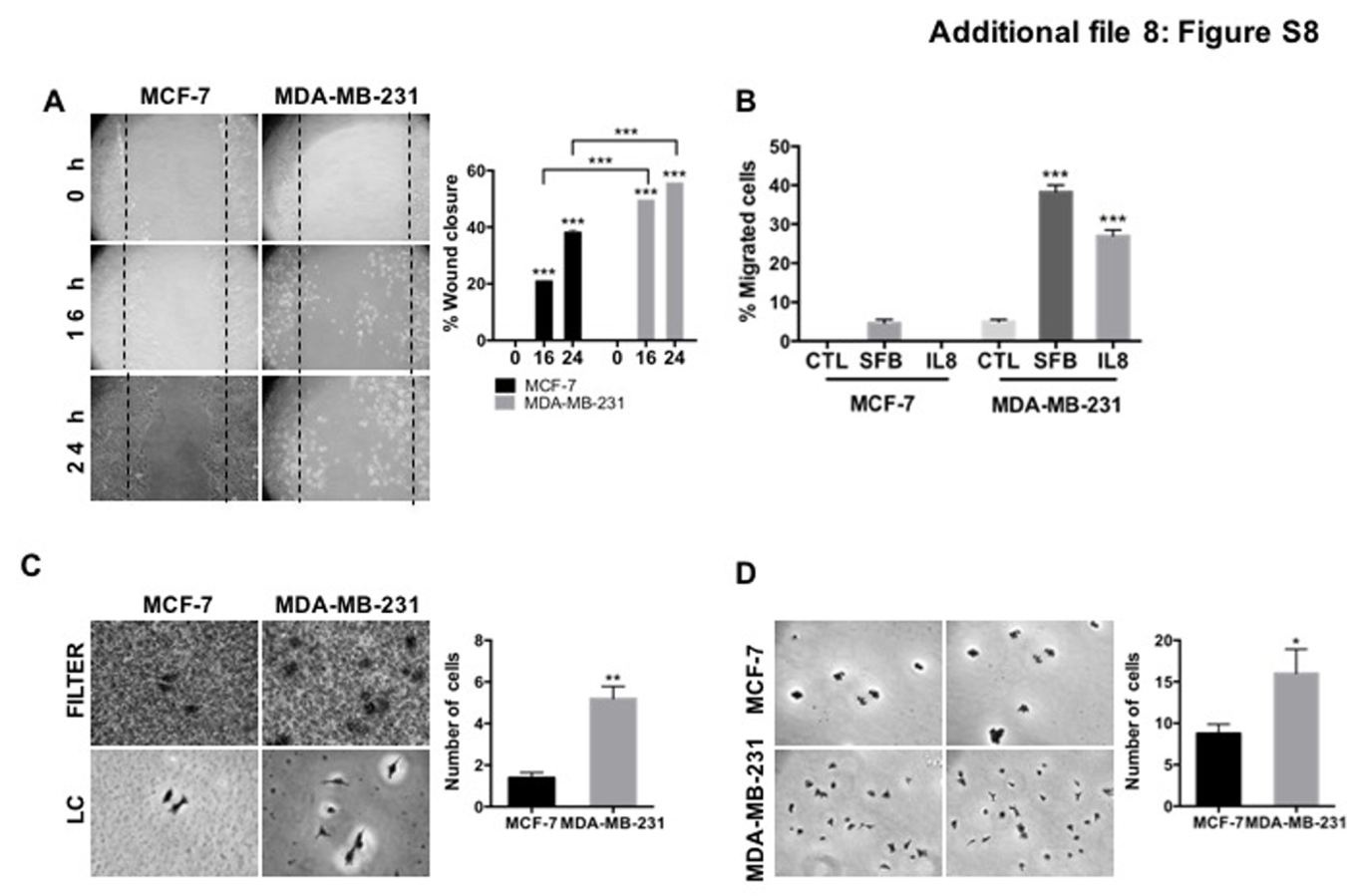

Supplement: Supplementary file 9 — MDA-MB-231 exhibits high migration capacity. (A) Wound healing assays were performed in MCF-7 and MDA-MB-231 cells. Representative micrographs (10×) were taken at 0, 16, and 24 h and showed an increased migration capacity of MDA-MB-231 cells (left). Black dotted line indicated the area of the wound. Quantification of wound healing assay from at least 10 images (right) by using the image J program. Error bars indicate SEM. (***p < 0.001) (n = 2). (B) Transwell migration assay performed in MCF-7 and MDA-MB-231 cells by using SFB (20%) or IL8 (50 ng/μl) as chemoattractants in the lower compartment. Error bars indicate SEM. (***p < 0.001). (C) Matrigel invasion assay; cells were allowed to invade during 48 h at 37 °C. Representative micrographs (20×) were taken from the upper panel (bottom surface of filters) and from the lower compartment (LC) and stained with violet Crystal. The histograms show the number of cells in the lower compartment. Error bars indicate SEM. (**p < 0.01) (n = 2). (D) Adhesion of MDA-MB-231 and MCF-7 cells to 96 multiwell plates coated with fibronectin. Representative micrographs (20×) were taken from the plates and stained with violet Crystal (left). The number of adherent cells was counted. Error bars indicate SEM. (*p < 0.05) (n = 3). (DOCX 646 kb) [file 12964_2017_172_MOESM9_ESM.docx]

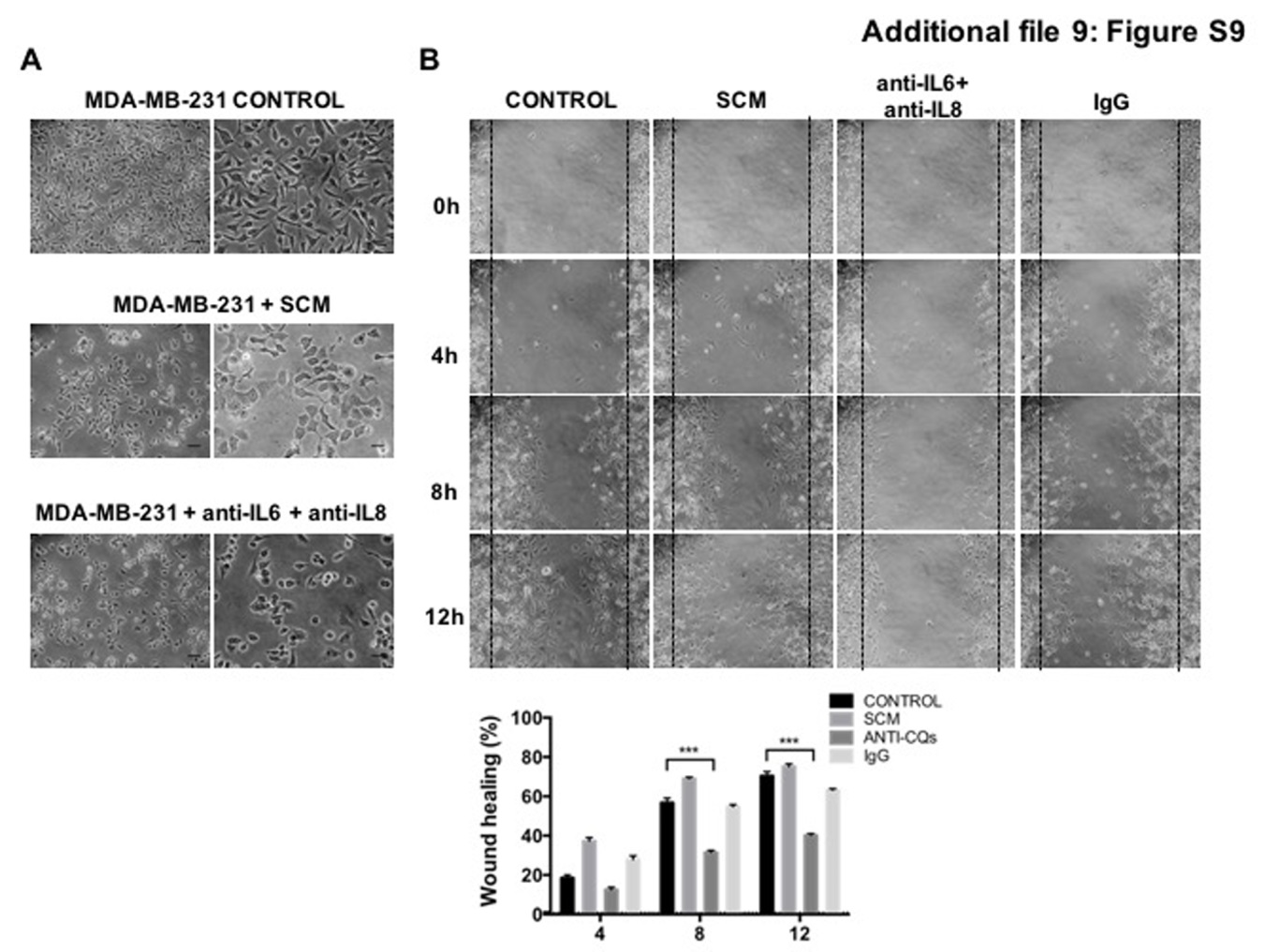

Supplement: Supplementary file 10 — Il6 and IL8 are relevant in the acquisition of functional properties of the EMT process in MDA-MB-231. MDA-MB-231 cells were treated or not (Control) with SCM or with 1 μg/ml of neutralizing monoclonal antibodies or IgG isotype control in RPMI medium without serum. (A) The morphological evaluation was done at 72 h after treatment. Representative images (10 and 20 ×) are shown. Scale bar, 10 μm. (B) Wound healing assays were performed in MDA-MB-231 cells. Representative micrographs (10×) were taken at 0, 4, 8 and 12 h and showed a decrease in migration capacity of MDA-MB-231 cells (upper). Black dotted line indicated the area of the wound. Quantification of wound healing assay from at least 10 images (bottom) by using the image J program. Error bars indicate SEM. (***p < 0.001) (n = 2). (DOCX 268 kb) [file 12964_2017_172_MOESM10_ESM.docx]

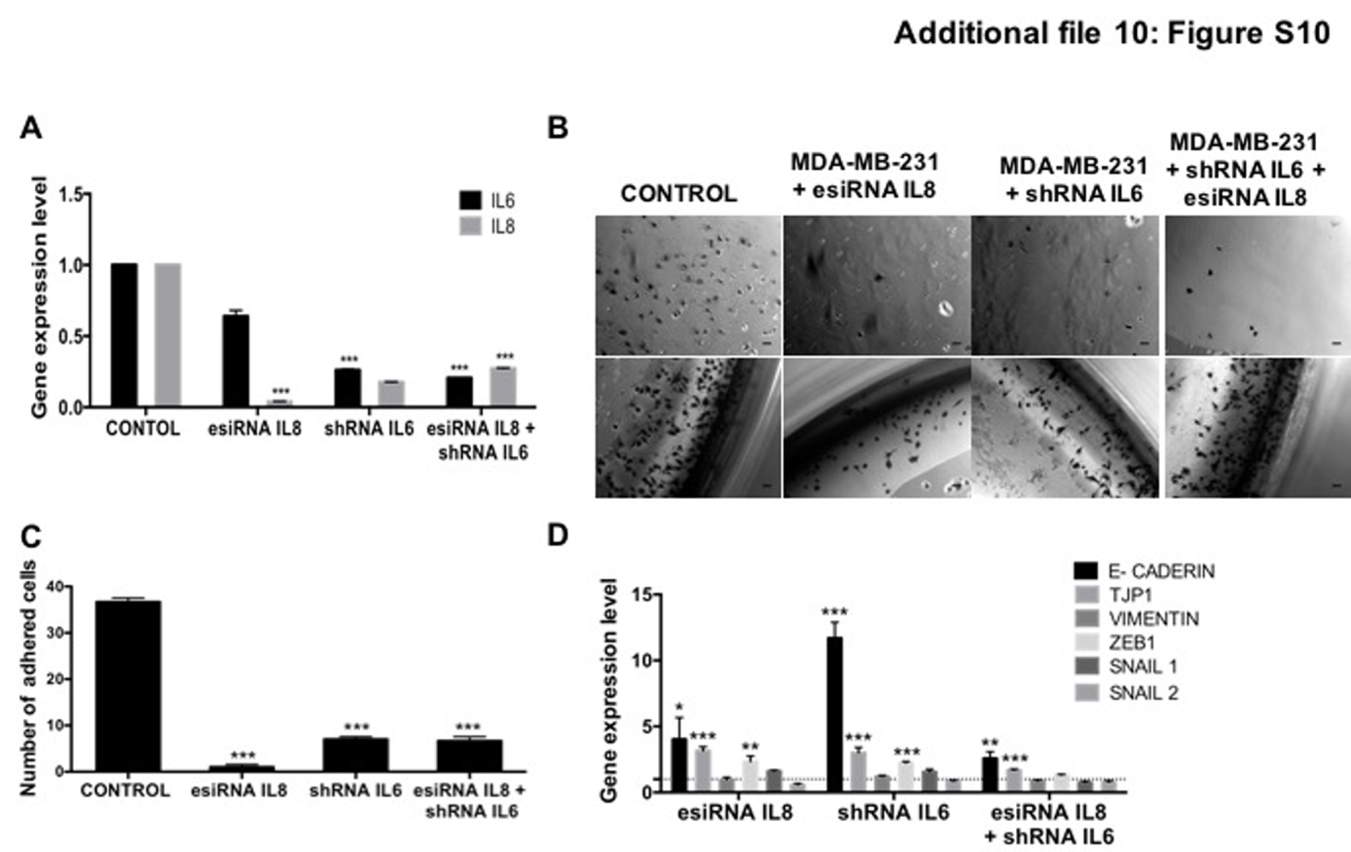

Supplement: Supplementary file 11 — Il6 and IL8 are important players in the acquisition of characteristics associated to EMT in MDA-MB-231. The knockdown of the pro-inflammatory cytokines was carried out by using esiRNA for IL8 and shRNA for IL6. The esiRNA-FLUC and shRNA Puro vector were used as control, respectively. (A) The decrease in the expression of these cytokines and (D) EMT markers was confirmed by qRT–PCR. The values were normalized to GADPH and relative to control cells (dotted lines). Error bars represent SEM. (*p < 0.05; **p < 0.01***p < 0.001). Adhesion of normal or IL6 and IL8 knockdown MDA-MB-231to 96 multiwell plates coated collagen. Representative micrographs (10×) were taken from the plates and stained with Crystal violet (B). The number of adherent cells was counted. Error bars indicate SEM. (***p < 0.001). (DOCX 578 kb) [file 12964_2017_172_MOESM11_ESM.docx]

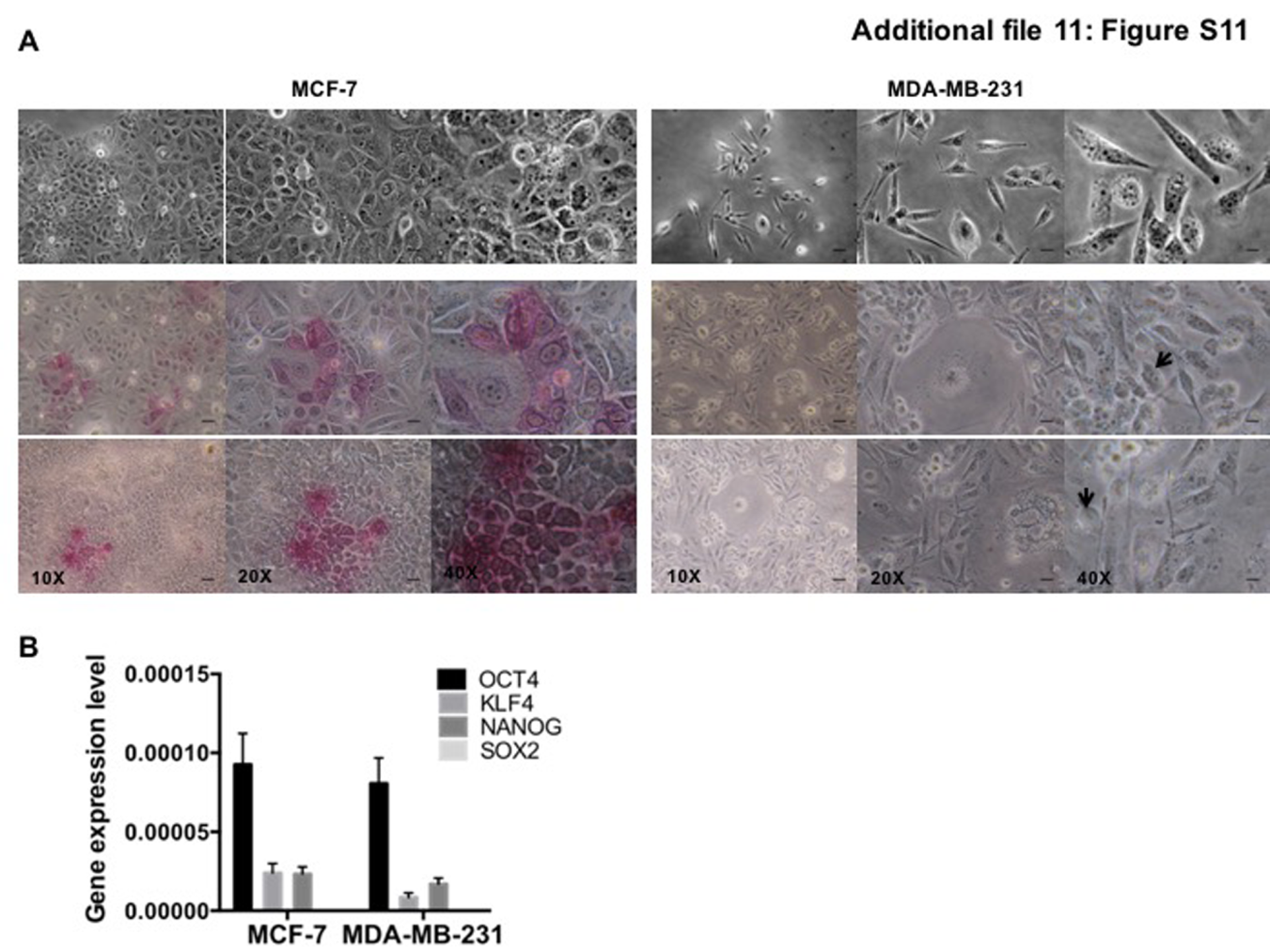

Supplement: Supplementary file 12 — MDA-MB-231 cannot differentiate to osteogenic lineage and are similar to MCF-7 in the expression of reprograming factors. (A) Differentiation was promoted towards the osteogenic lineage by using specific induction medium. Differentiation was evaluated after 10 days with specific staining (APL activity). Representative images are shown (10, 20 and 40 ×). Scale bar, 10 μm. Cells without induction medium were used as a control. (B) Gene expression levels of reprogramming-associated transcription factors were evaluated by qRT–PCR. The values were normalized to GADPH. Error bars represent SEM. (DOCX 1247 kb) [file 12964_2017_172_MOESM12_ESM.docx]
